# Supplementary material for: Manual Uterine Aspiration Simulation for Emergency Medicine Learners
Source: MedEdPORTAL. 2024 Nov 11;20:11469. doi: 10.15766/mep_2374-8265.11469 (PMC11551269; doi:10.15766/mep_2374-8265.11469)
Supplement: Supplementary file 1 — MUA Model Preparation.docxStation Setup and Supplies.docxMUA Lecture.pptxMUA Video Demonstration.m4vFacilitator Guides.docxProcedure Checklist.docxLearner Survey.docxFacilitator Survey.docx [file mep_2374-8265.11469-s001.zip › A. MUA Model Preparation.docx]

**Manual Uterine Aspiration Simulation Model**

**Assembly/Preparation Instructions**

How to use this appendix: This appendix should be used prior to the training to guide you in model building and preparation. All items will need to be acquired and the models built prior to simulation day. The number of models you will need will vary depending on number of learners. Each model takes about 4 minutes to make.

This high-fidelity, low-resource, non-perishable, and reproducible hands-on model for training medical providers was developed by Dr. Meg O’Reilly and colleagues from the Department of Obstetrics and Gynecology at Oregon Health Science University. This PDF was adapted with Dr. O’Reilly’s permission, based on her instructional video on MUA model assembly. A papaya model can also be used if preferred.

**Uterine Model Supplies (pictured below):**

*Makes 1 uterine model.*

- 3 pink or red balloons
  - 1 with a slit cut at top and the balloon neck cut off
  - 1 with balloon neck cut off
  - 1 with top cut off
- 1 pool noodle with hollow core
- 1 Scünci Super Bandz Evolution hair elastic
- 1 Gaiam stress relief ball (any firmness will work well)
- 2, 2-inch wide segments of adhesive Velcro strips (rough side only)

**Imitation “Products of Conception” Supplies:**

- Tapioca small pearls
- Red food coloring
- Gelatin or red-colored Jello packets
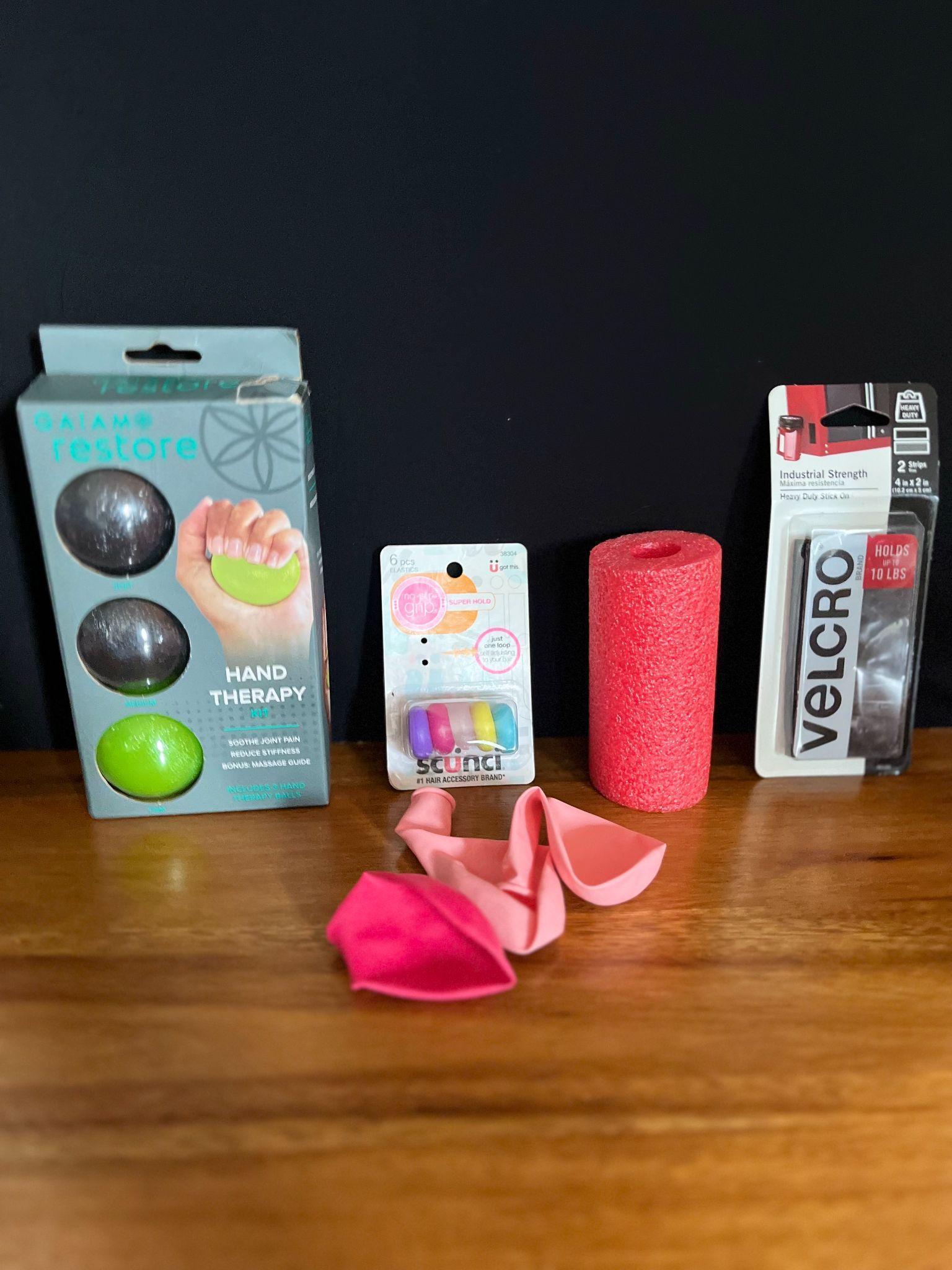


**(**Author owned image)

**Imitation “Products of Conception” Preparation Instructions:**

*Plan to prepare this at least 1 day before the simulation*

1. Make gelatin or Jello as described on the package. Then, add extra water (amount will vary depending on amount of gelatin used)
2. If the mixture is clear, add the red food coloring. If a red Jello mixture was used, you can forgo this step.
3. Add tapioca balls to the gelatin/water mixture
4. Place mixture in the fridge to cool and congeal. Check on the mixture every couple of hours and stir it up. The goal is not to make a block of Jello, but rather, a heterogenous mixture (as pictured below). You may need to add more boiling water during these mixing breaks if the Jello mixture has become too firm or homogenous. This can rest in the fridge overnight or for a couple of nights before use with the models


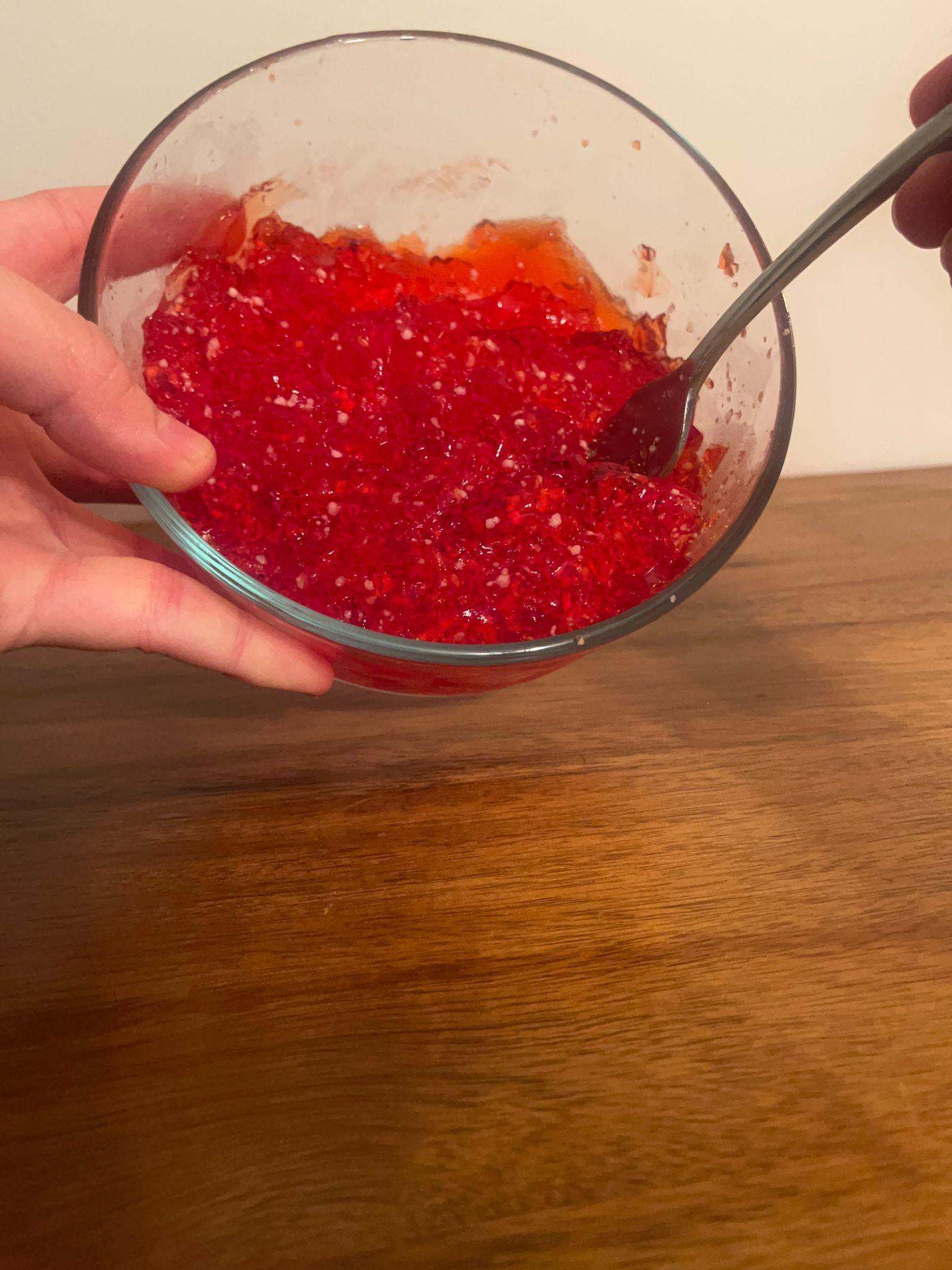


**Note:** The goal is to make the mixture thick enough to resemble products of conception but thin enough to be aspirated from the aspirator model used in the simulation. When in doubt, thinner is better. When left out of the fridge, the mixture will naturally start to thin as it warms.

**Uterine Model Assembly Instructions:**

1. Take a 6-foot hollow pool noodle in any color and cut it into 6-inch segments.
2. Carve out one side of the pool noodle opening to create a tapering effect within the cavity. Be careful that the surrounding foam does not become too thin. You should only carve out that side to halfway down the segment.


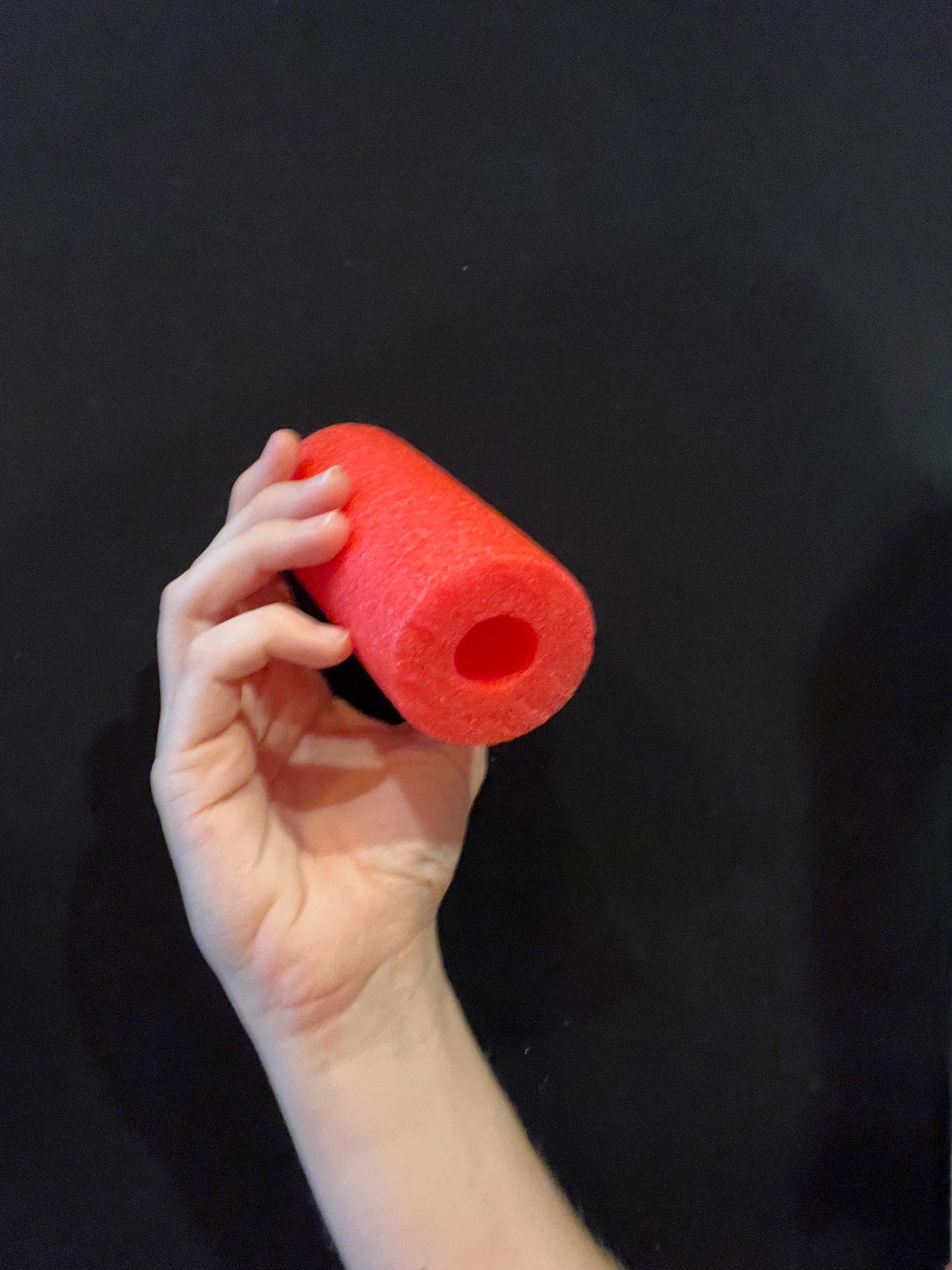


1. Cut two strips of adhesive Velcro to the length of the pool noodle segment.


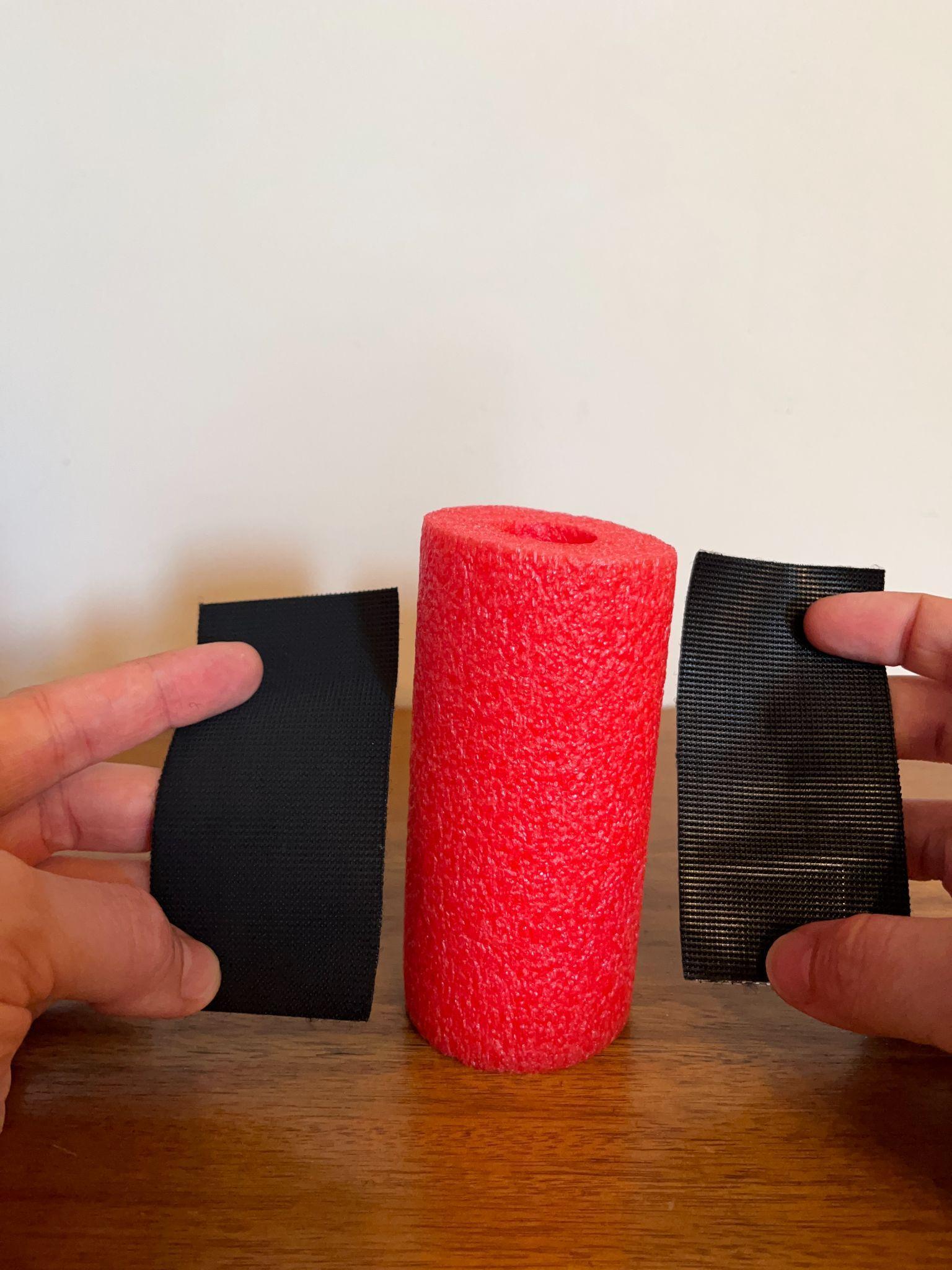


1. Remove the paper backing from the adhesive Velcro. Fold the piece of Velcro in half lengthwise (rough sides together). Feed this piece of Velcro through the opening of the pool noodle segment.


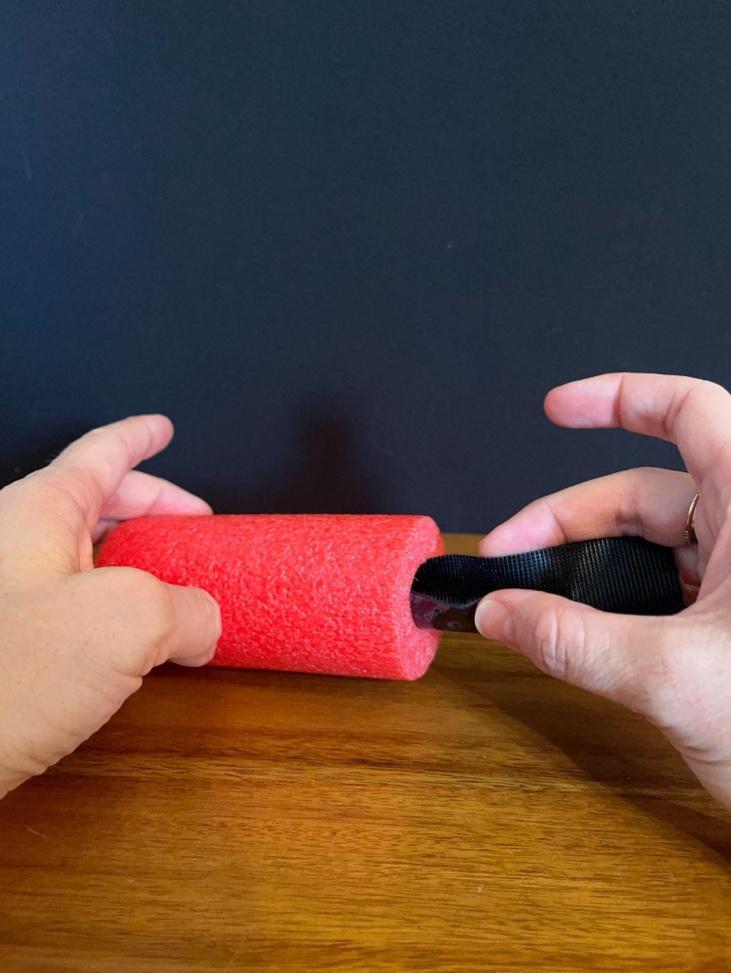


A partner can use hemostats to grab the folded ends on the other side of the pool noodle segment and feed it the rest of the way through. You want to be sure the inside of the Velcro doesn’t stick to the interior foam until it is positioned properly inside the lumen of the pool noodle.

1. Repeat Step 5 on the other side of the pool noodle segment, so that the entire inside of the segment is lined with the rough Velcro. Press the Velcro down firmly on the inside. This portion simulates the uterine cavity and will give a gritty consistency, similar to that felt with an empty uterus.


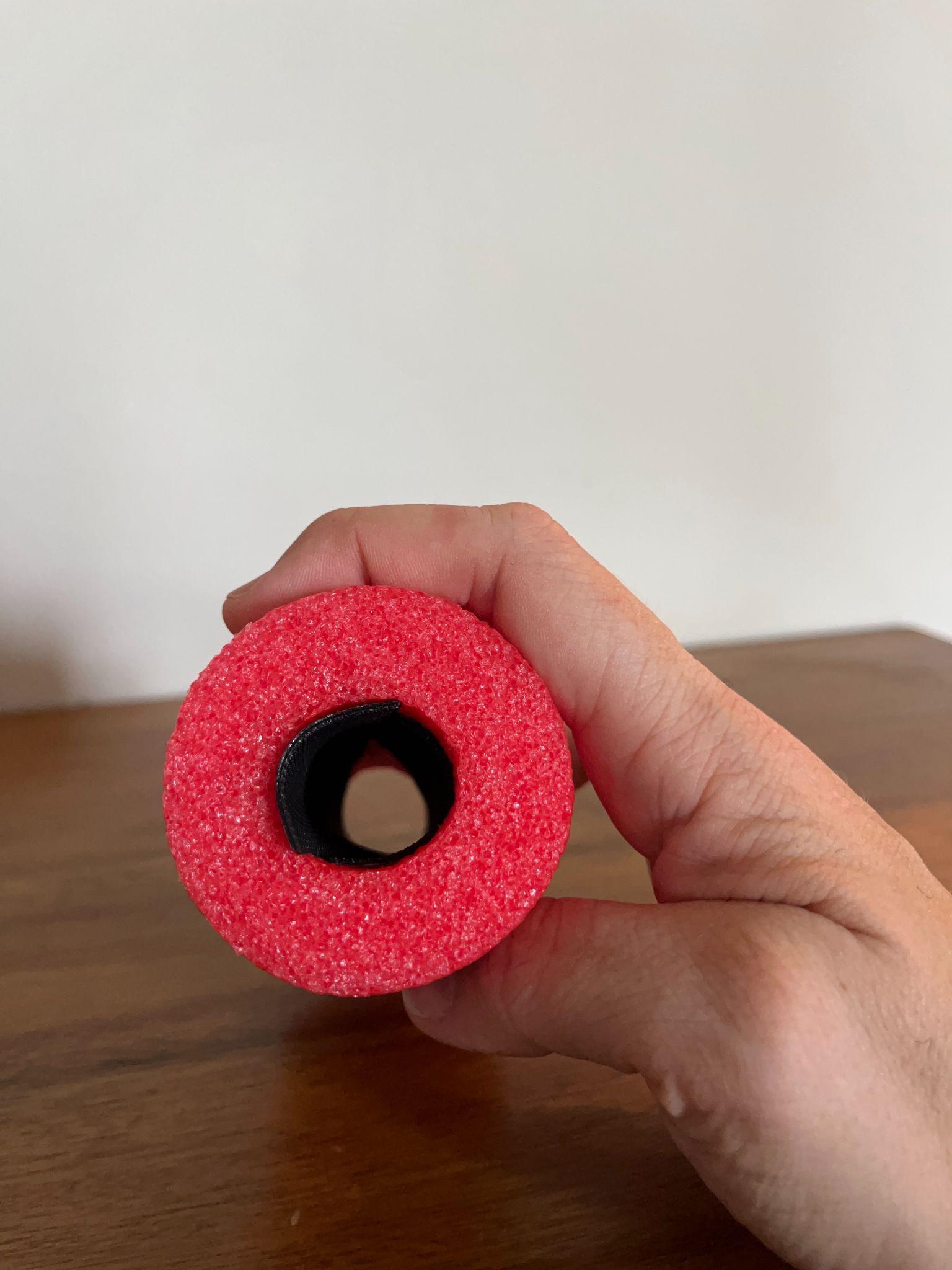


1. Take a stress ball and cut it in half with sharp scissors. The cut half of the stress ball will simulate the internal cervical os, through which the cannula will pass


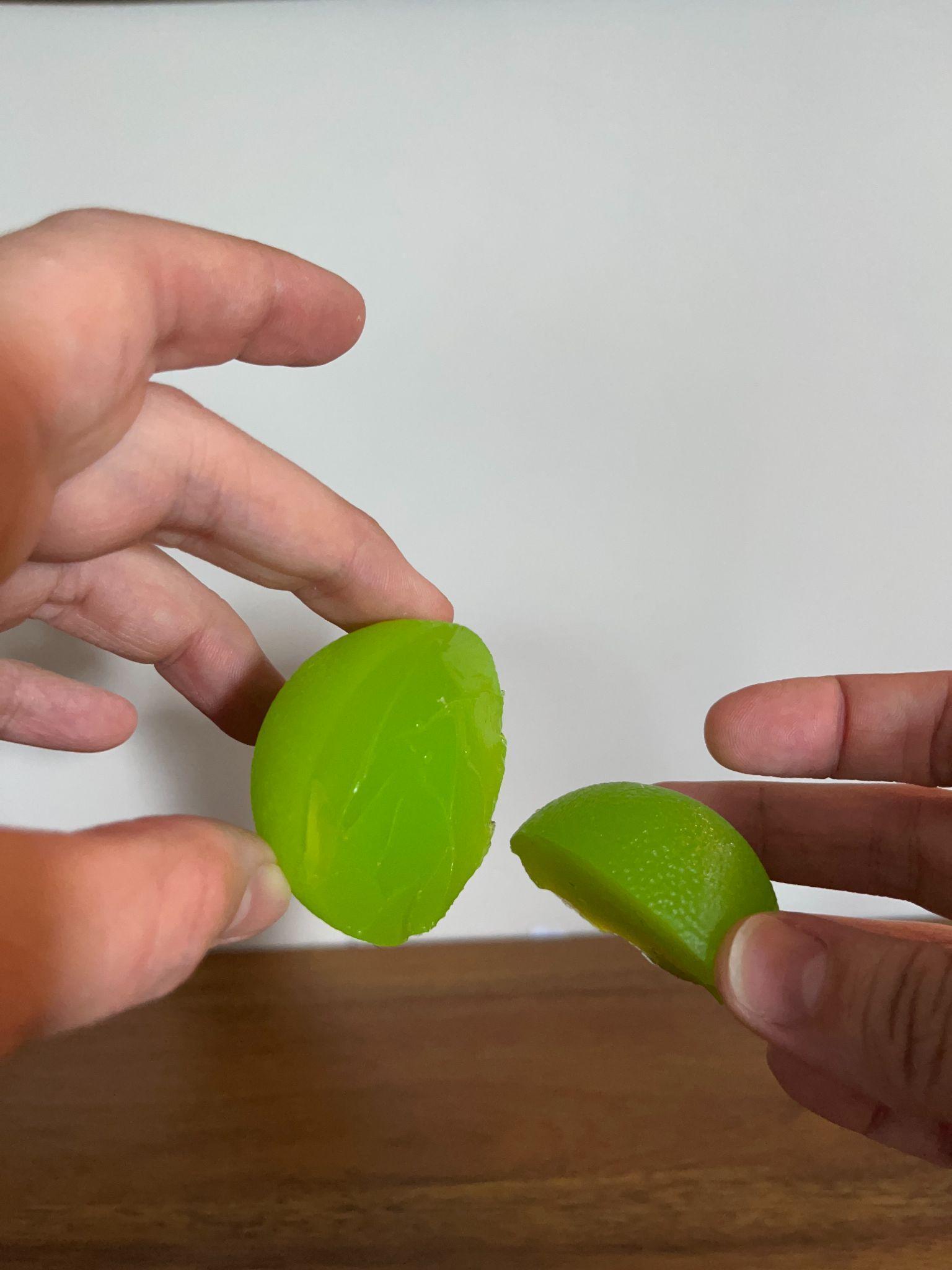


1. Cut out a divot on the rounded surface of your half stress ball (as shown in the completed example next to the halved stress ball below). This divot will allow you to seat a hairband, which will simulate the external cervical os. Make sure that your hairband representing the cervix will sit within the carved-out surface you have created within the stress ball.


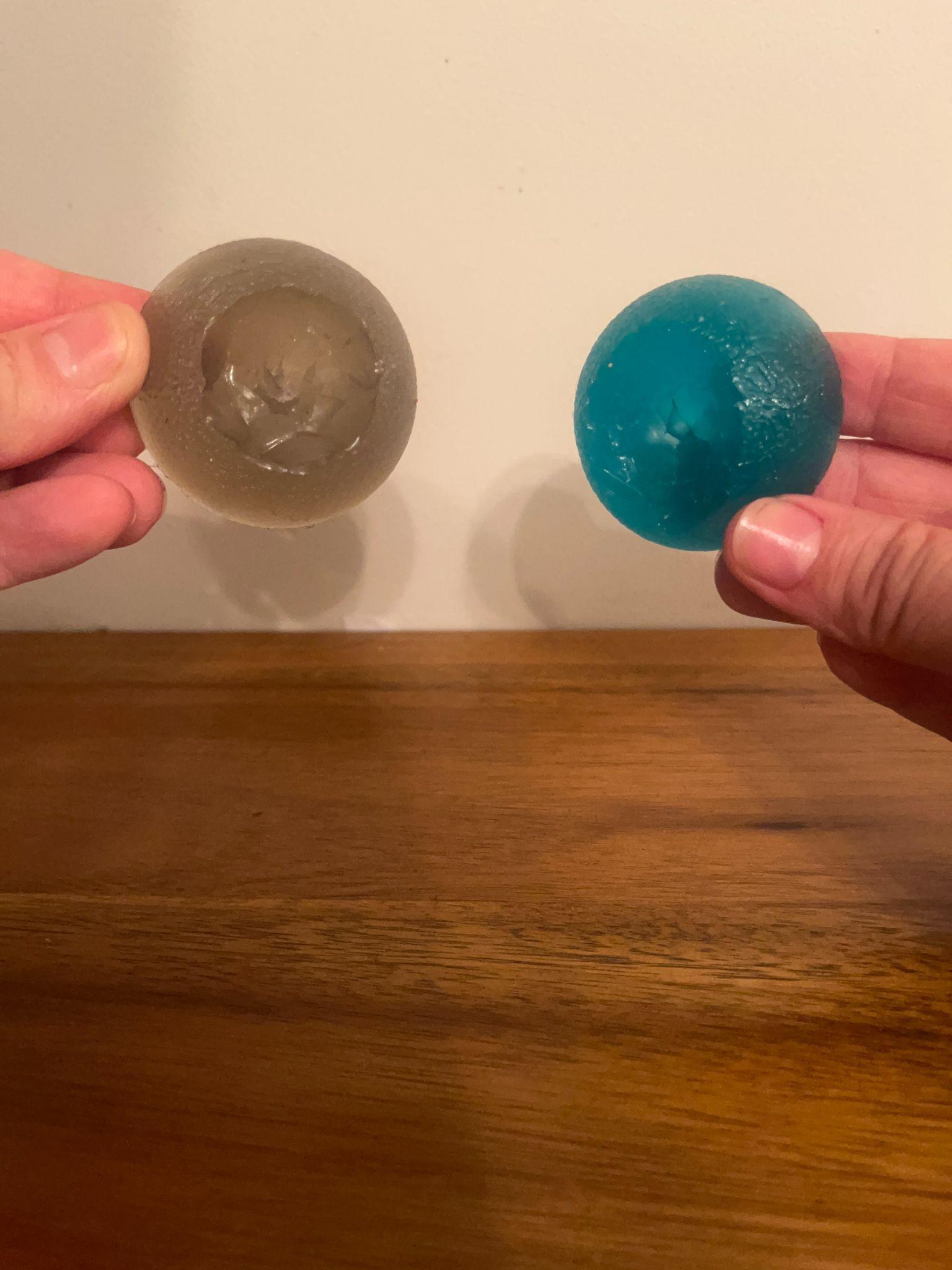


1. Drive your scissors through the center of the halved stress ball in order to create a tunnel for your cannula to advance.
2. Take one balloon and advance the tying end/neck of the balloon through the hairband as shown. Wrap the end of the balloon around the hairband.


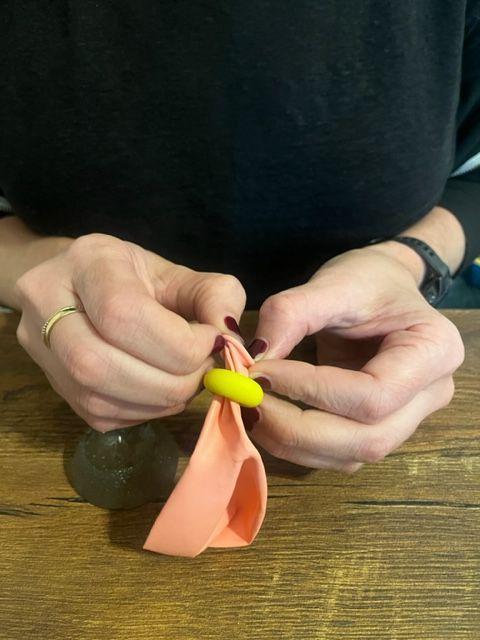

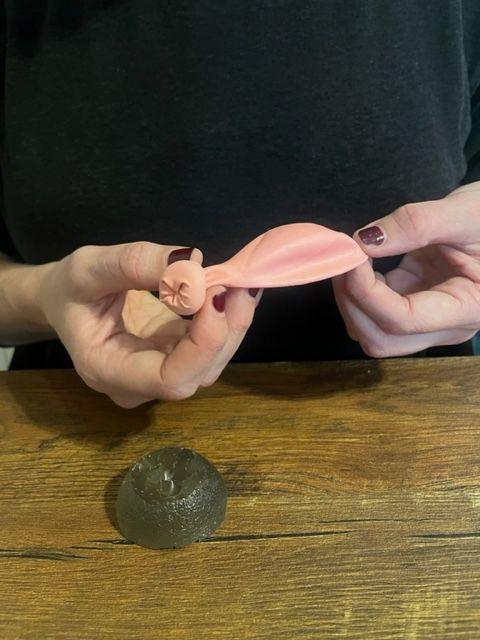


1. Advance the non-tying end of the balloon through the halved stress ball as shown, so that the balloon-wrapped hairband ultimately sits inside of the groove on the halved stress ball.


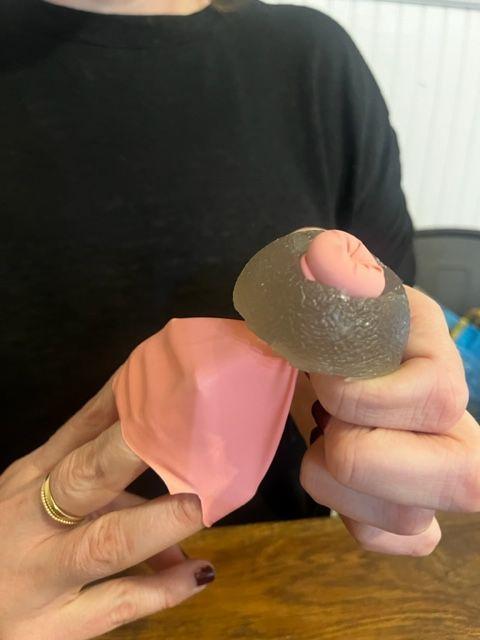


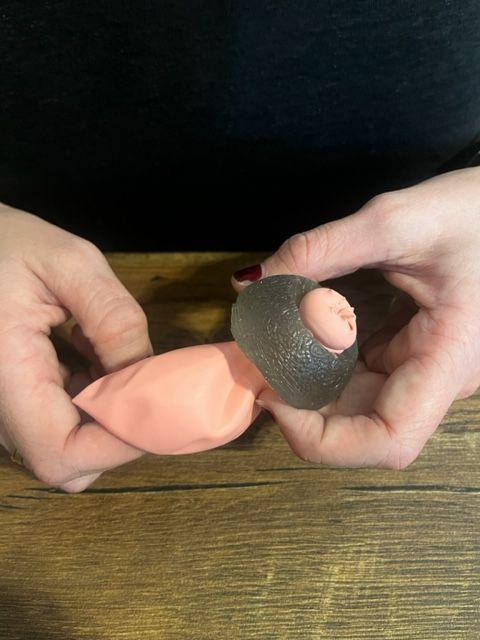


1. Cut the top of the balloon off in a straight line.
2. Take the completed foam segment, and with the larger opening facing up, set it on a surface as a partner holds it in place. Stretch the cut balloon down over the segment so that the stress ball and hairband apparatus sit on top.
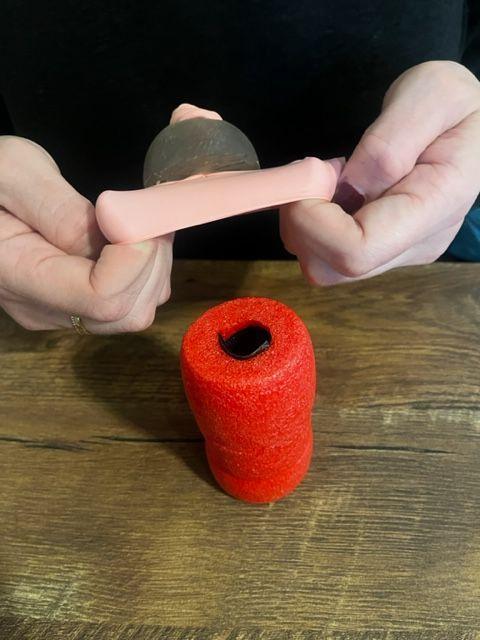


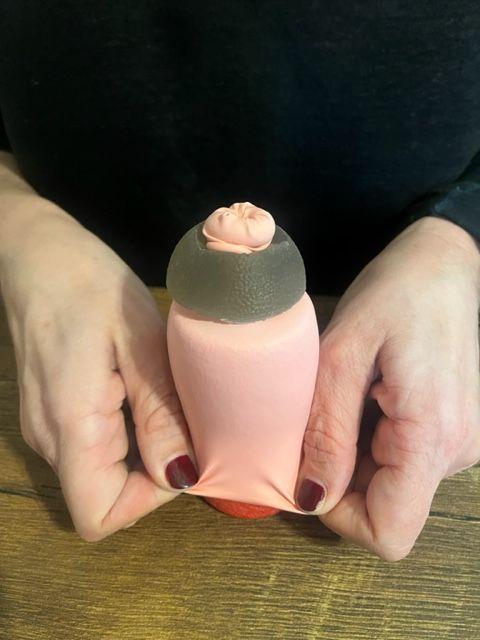

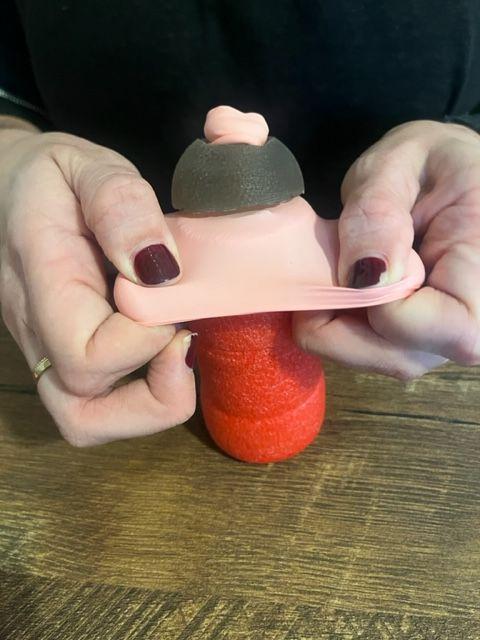


1. Take another balloon and cut a small slit in the top. Make sure it is not too big, or else the hair band representing the cervix may poke through the slit during the model use. Cut the neck of the balloon off.


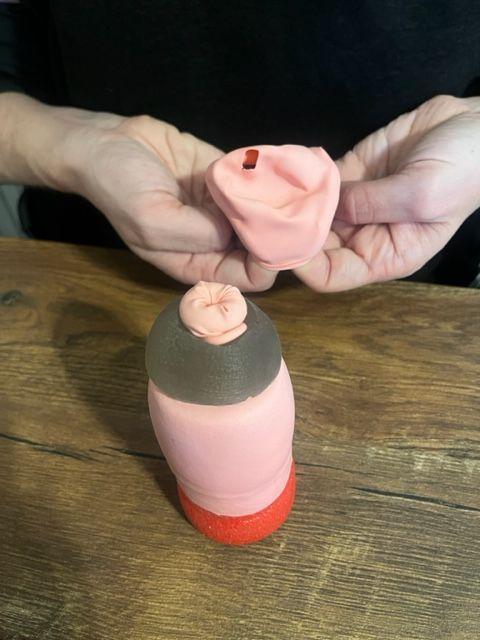


1. Stretch this balloon down over your model to secure the stress ball and hairband apparatus in place, taking care to center the fenestration over the hairband opening.


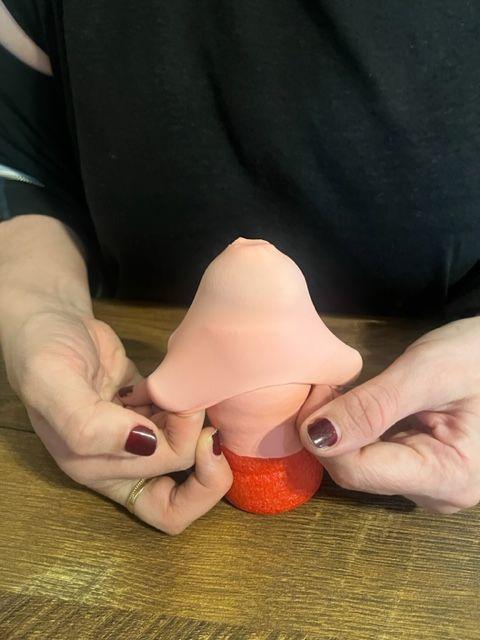

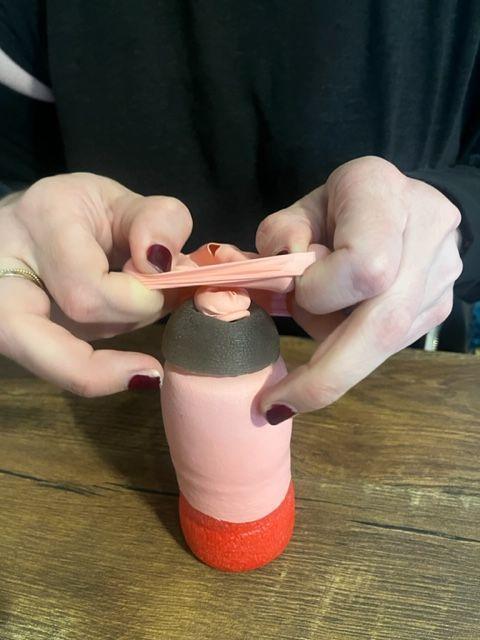


1. From the open side of the pool noodle segment, fill the model with imitation products of conception.
2. Seal the segment with another balloon with the neck cut off and stretch it down over the back of the segment. To keep the balloon coverings in place, secure the balloons with a wide rubber band. Your model is now ready for use.


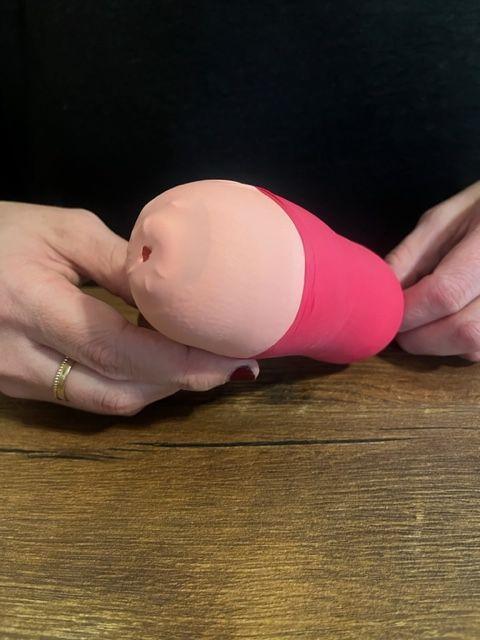


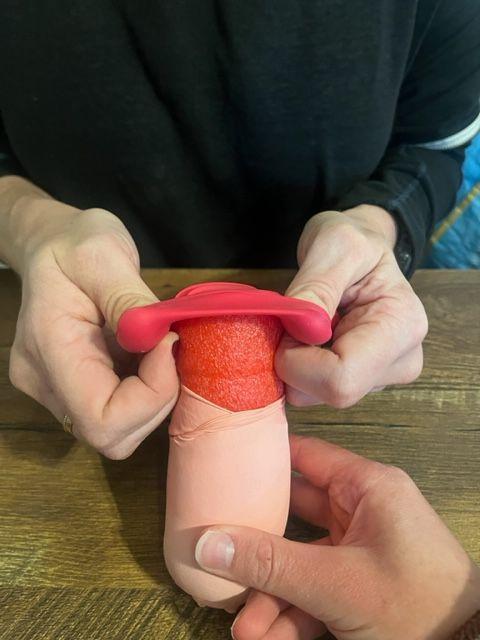


**Note:** Steps 1-15 can be prepared ahead of time. However, if the balloons are left stretched over the pool noodle for multiple days, the foam will indent. We recommend preparing the component parts in advance and then assembling the model the day before or day of your simulation.
