# Supplementary material for: Manual Uterine Aspiration Simulation for Emergency Medicine Learners
Source: MedEdPORTAL. 2024 Nov 11;20:11469. doi: 10.15766/mep_2374-8265.11469 (PMC11551269; doi:10.15766/mep_2374-8265.11469)
Supplement: Supplementary file 1 — MUA Model Preparation.docxStation Setup and Supplies.docxMUA Lecture.pptxMUA Video Demonstration.m4vFacilitator Guides.docxProcedure Checklist.docxLearner Survey.docxFacilitator Survey.docx [file mep_2374-8265.11469-s001.zip › B. Station Setup and Supplies.docx]

Appendix B: Simulation Set-Up and Supplies

How to use this appendix: Use this as a reference for setting up your hands-on training including the supplies needed at each station. We recommend starting to set up about 25 minutes before the simulation starts to ensure stations are prepared. Stations 1 and 2 will each take about 10 minutes for the learner, and Station 3 will take about 15 minutes.

**Station I: Cervical Anesthesia**

**Set-Up:**
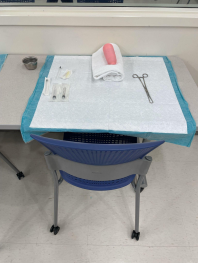


**(author owned image)**

**Supplies**

● Tenaculum

● 10cc syringe

● 22-gauge spinal needle

● One uterine model for the entire session with 1-2 backup models

Appendix B: Simulation Set Up and Supplies **Station II: Cervical Dilation**

**Set-up**
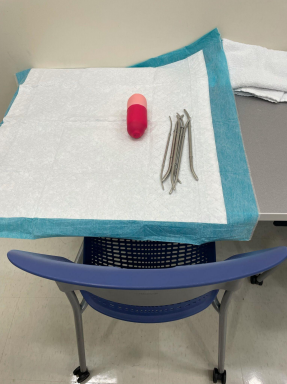


**(author owned image)**

**Supplies**

● One uterus model for every 3-4 learners

● Cervical dilators

Appendix B: Simulation Set Up and Supplies

**Station III: Full MUA Procedure**

**Set-up**


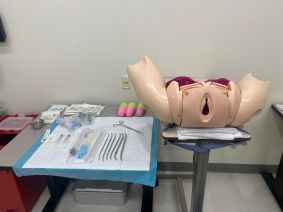


**(author owned image)**

**
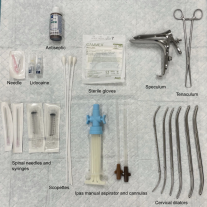
(author owned image)**

**Supplies:**

● Sterile gloves

● Antiseptic

● 18-gauge needle

● (2) 10cc syringes

● (2) 22-gauge spinal needles

● Speculum

● Tenaculum

● Scopettes

● Cervical dilators

● Manual vacuum aspirator and cannulas

● Hemipelvic model
