# Supplementary material for: Manual Uterine Aspiration Simulation for Emergency Medicine Learners
Source: MedEdPORTAL. 2024 Nov 11;20:11469. doi: 10.15766/mep_2374-8265.11469 (PMC11551269; doi:10.15766/mep_2374-8265.11469)
Supplement: Supplementary file 1 — MUA Model Preparation.docxStation Setup and Supplies.docxMUA Lecture.pptxMUA Video Demonstration.m4vFacilitator Guides.docxProcedure Checklist.docxLearner Survey.docxFacilitator Survey.docx [file mep_2374-8265.11469-s001.zip › C. MUA Lecture.pptx]

## Slide 1
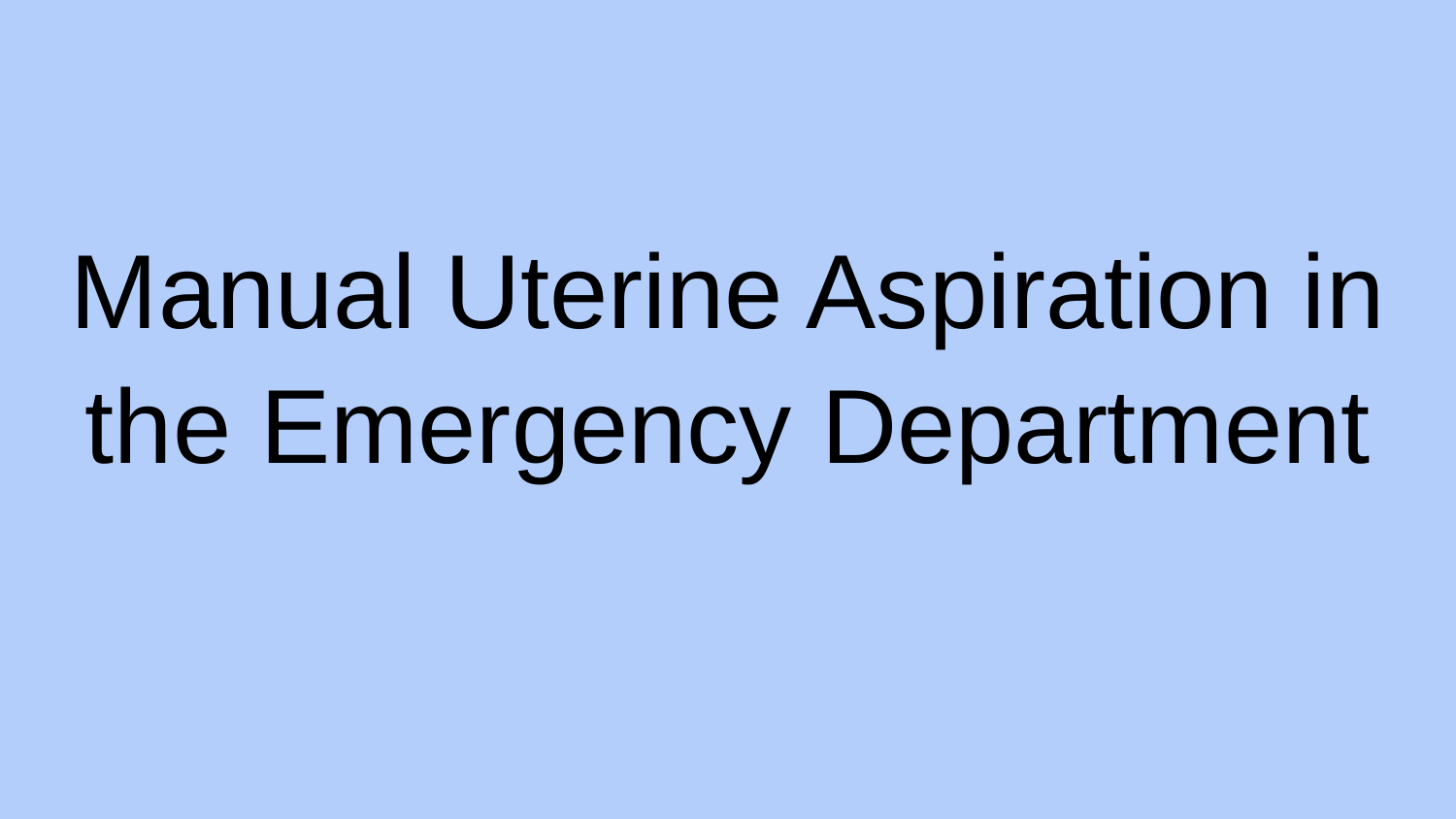

# Manual Uterine Aspiration in the Emergency Department

## Slide 2
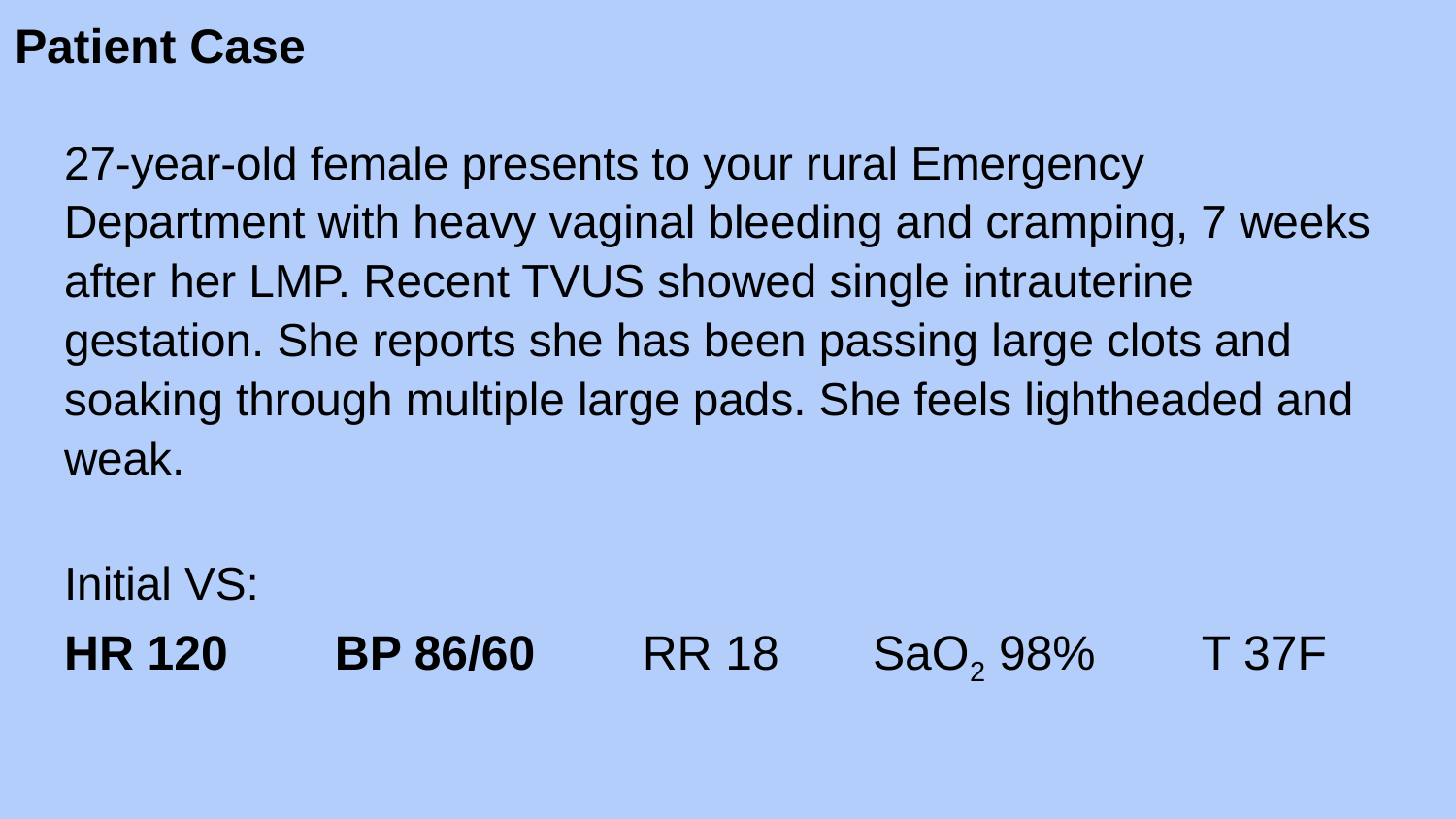

# Patient Case
27-year-old female presents to your rural Emergency Department with heavy vaginal bleeding and cramping, 7 weeks after her LMP. Recent TVUS showed single intrauterine gestation. She reports she has been passing large clots and soaking through multiple large pads. She feels lightheaded and weak.
Initial VS:
HR 120 BP 86/60 RR 18 SaO2 98% T 37F

## Slide 3
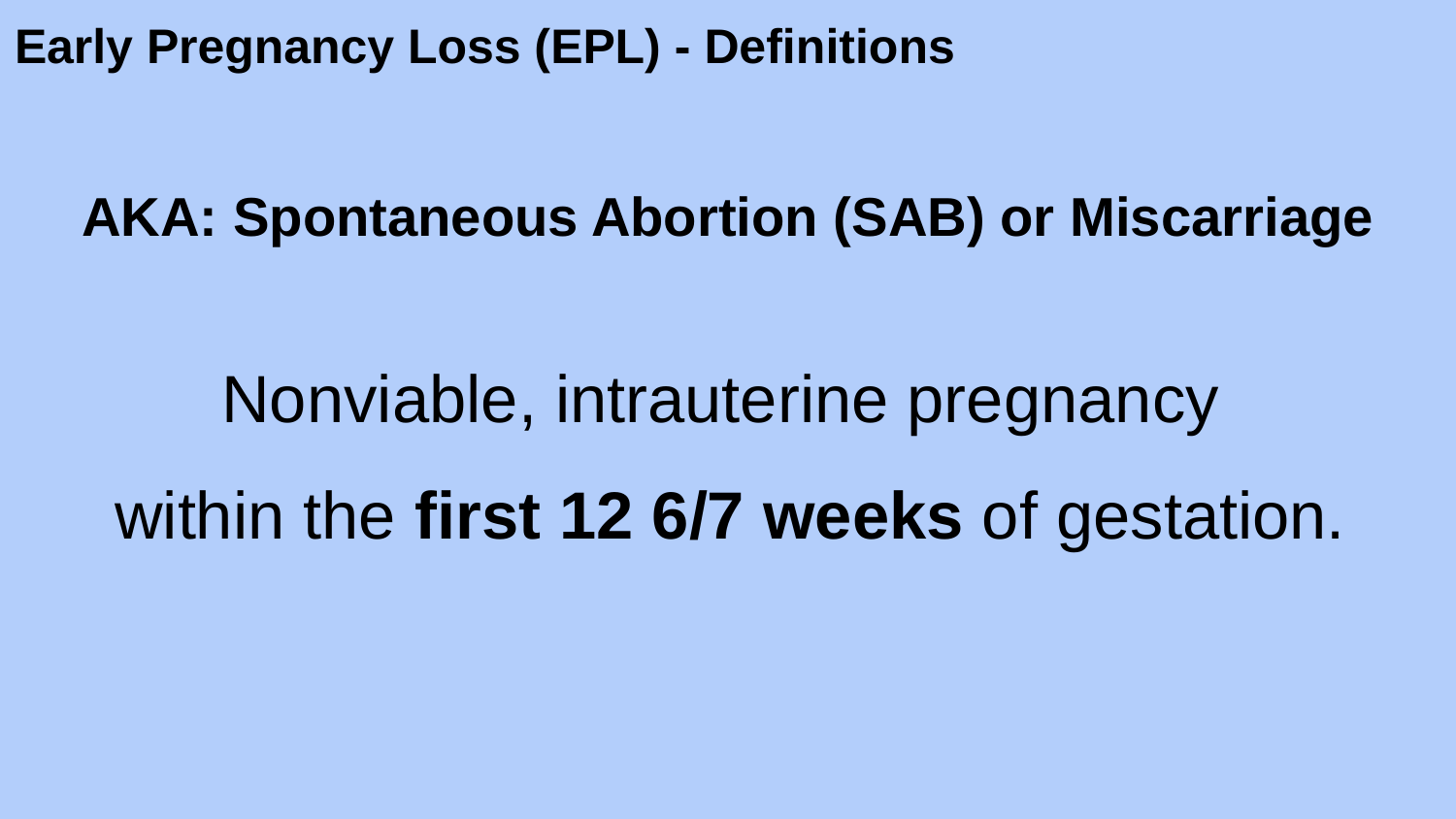

# Early Pregnancy Loss (EPL) - Definitions
AKA: Spontaneous Abortion (SAB) or Miscarriage
Nonviable, intrauterine pregnancy
within the first 12 6/7 weeks of gestation.

## Slide 4
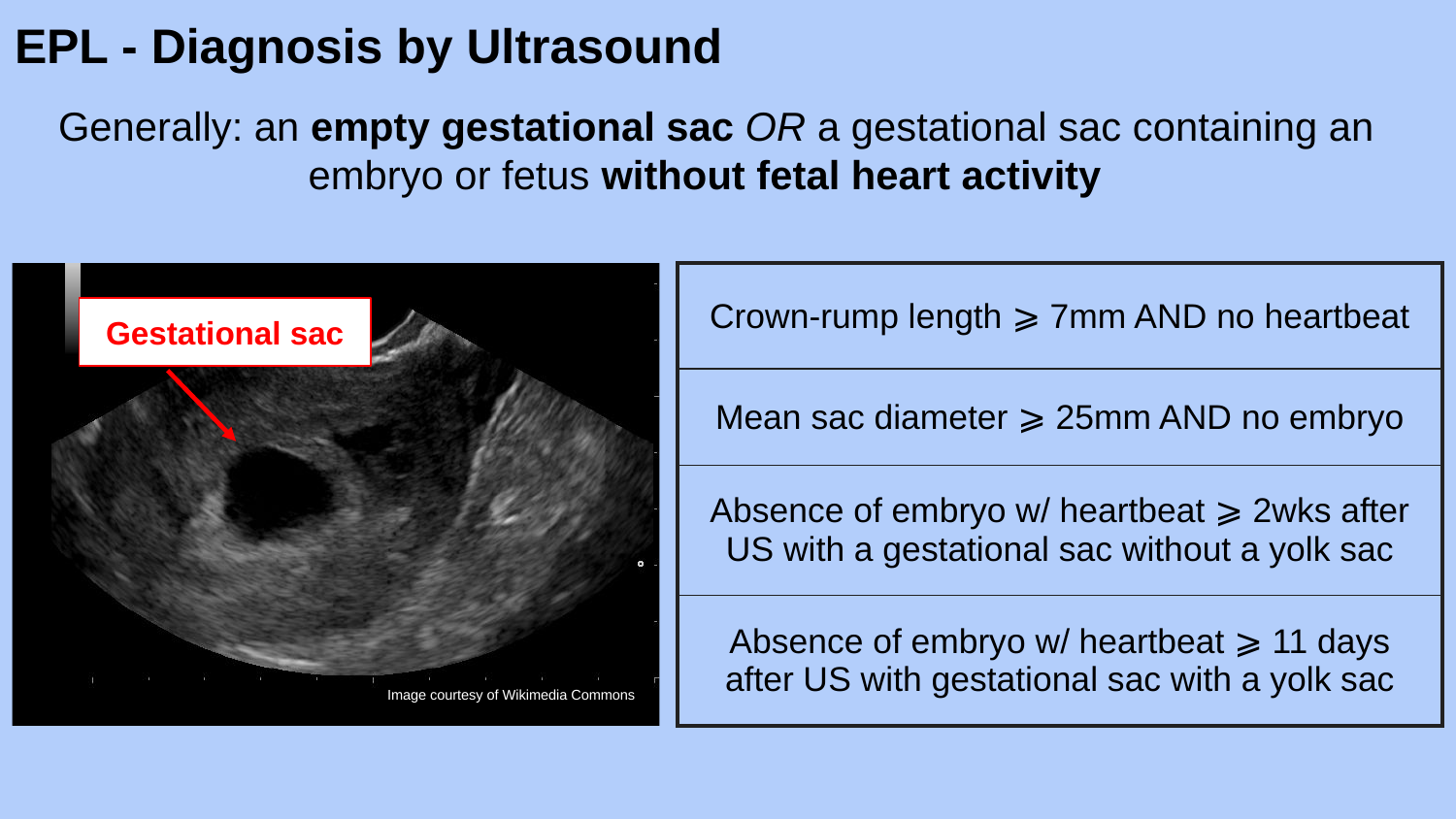

# EPL - Diagnosis by Ultrasound
 Generally: an empty gestational sac OR a gestational sac containing an embryo or fetus without fetal heart activity
Image courtesy of Wikimedia Commons
Gestational sac
| Crown-rump length ⩾ 7mm AND no heartbeat |
| --- |
| Mean sac diameter ⩾ 25mm AND no embryo |
| Absence of embryo w/ heartbeat ⩾ 2wks after US with a gestational sac without a yolk sac |
| Absence of embryo w/ heartbeat ⩾ 11 days after US with gestational sac with a yolk sac |

## Slide 5
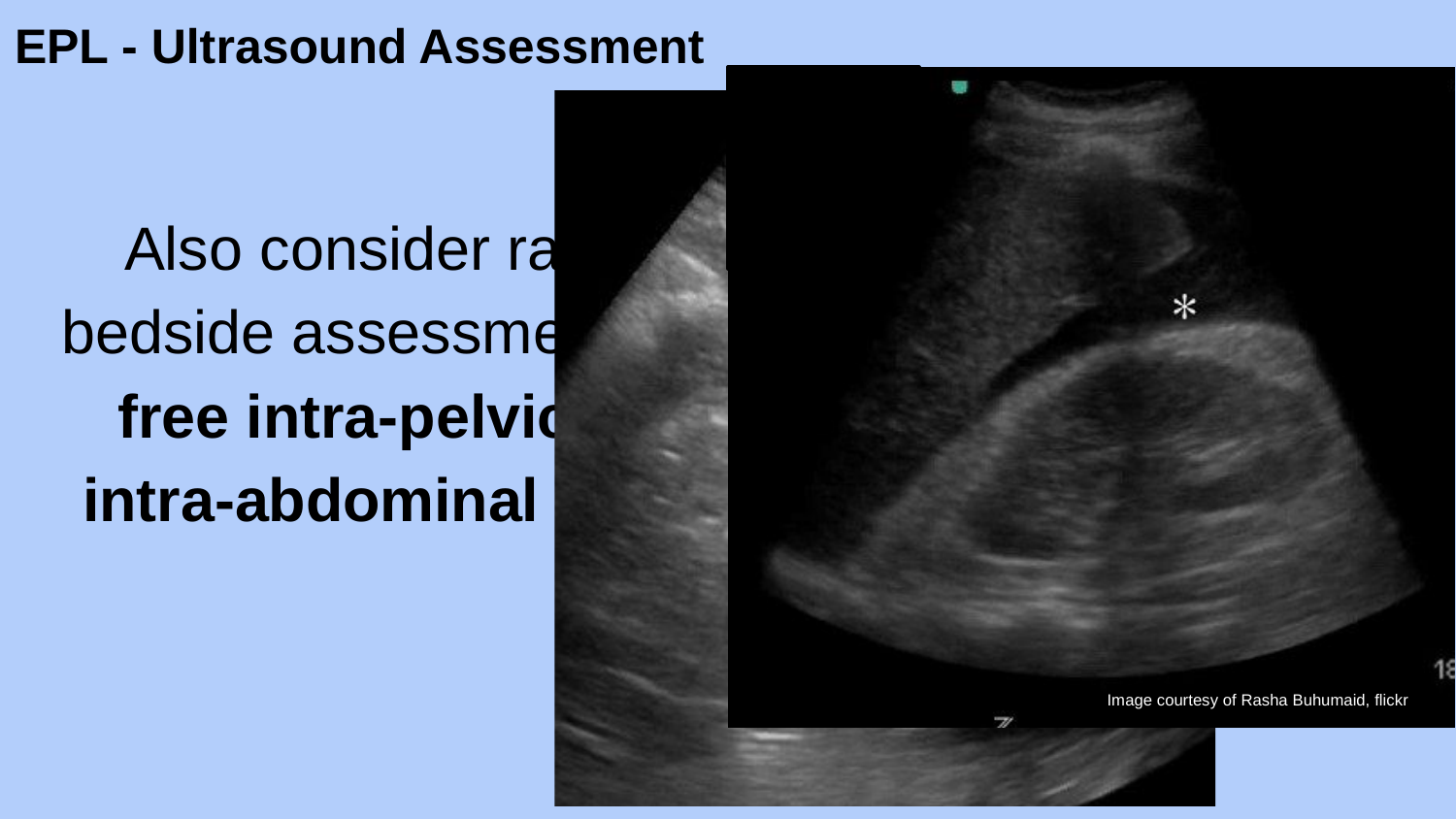

# EPL - Ultrasound Assessment
Also consider rapid bedside assessment for free intra-pelvic or intra-abdominal fluid
Image courtesy of Rasha Buhumaid, flickr

## Slide 6
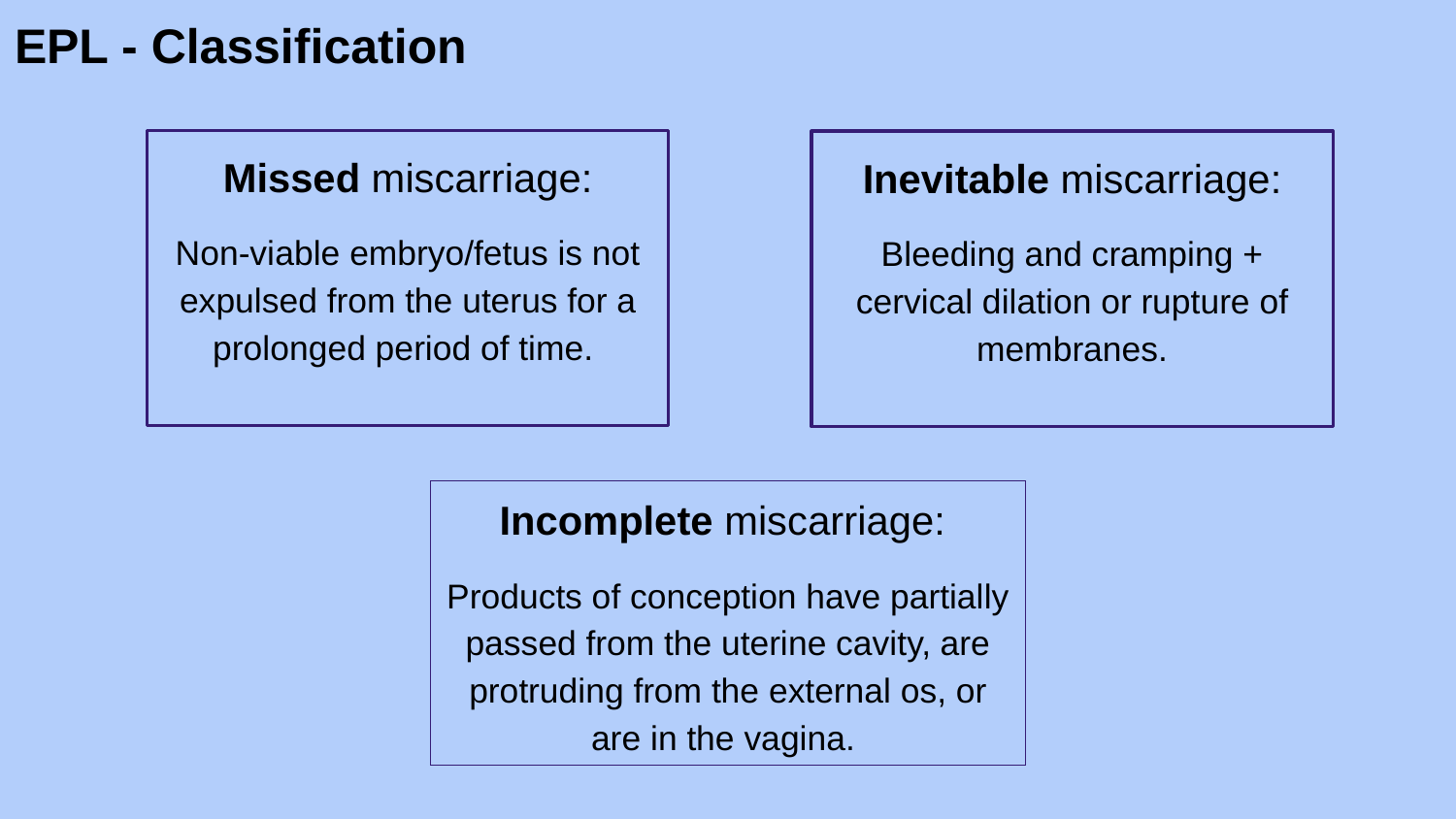

# EPL - Classification
Missed miscarriage:
Non-viable embryo/fetus is not expulsed from the uterus for a prolonged period of time.
Inevitable miscarriage:
Bleeding and cramping + cervical dilation or rupture of membranes.
Incomplete miscarriage:
Products of conception have partially passed from the uterine cavity, are protruding from the external os, or are in the vagina.

## Slide 7
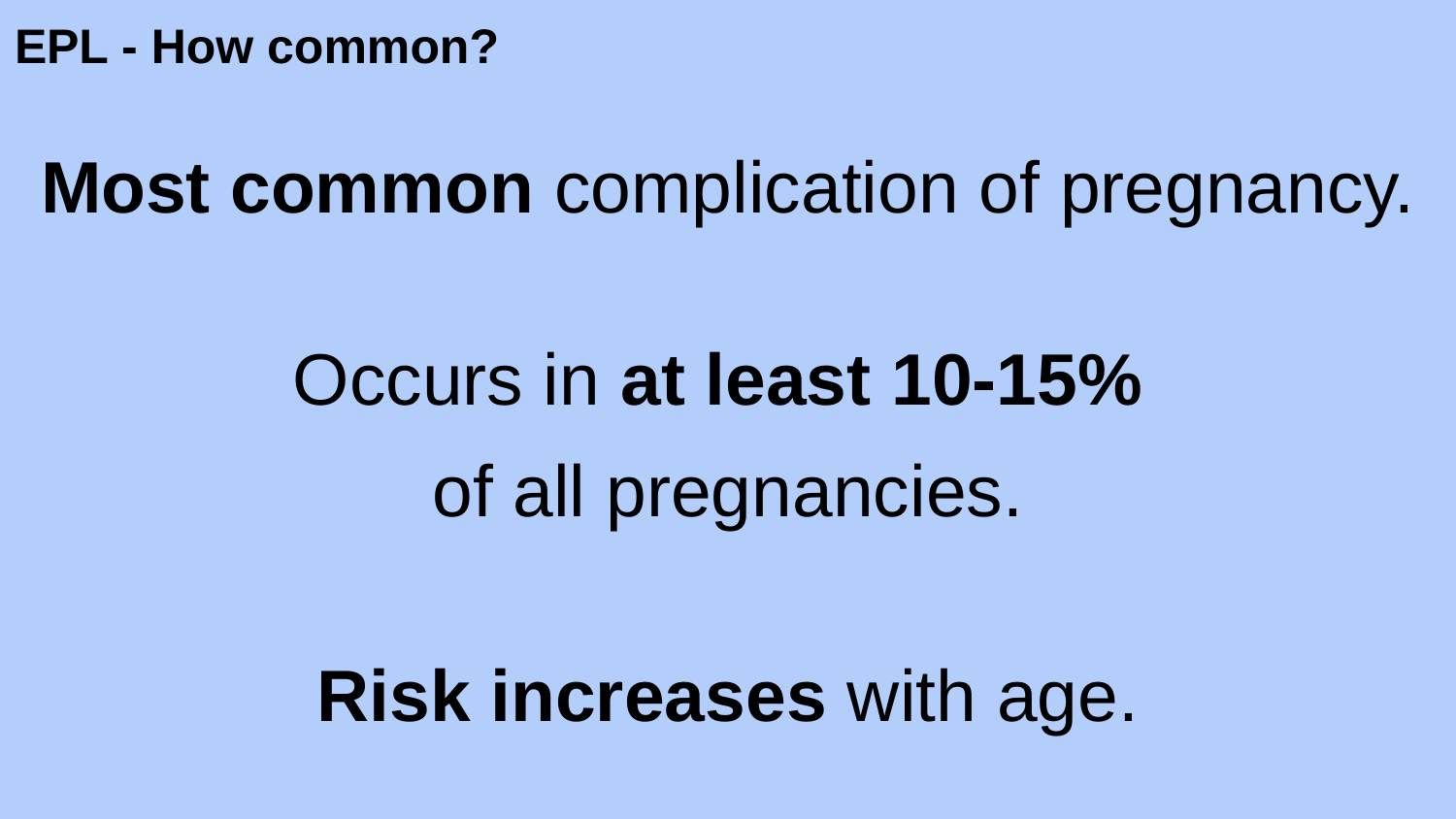

# EPL - How common?
Most common complication of pregnancy.
Occurs in at least 10-15%
of all pregnancies.
Risk increases with age.

## Slide 8
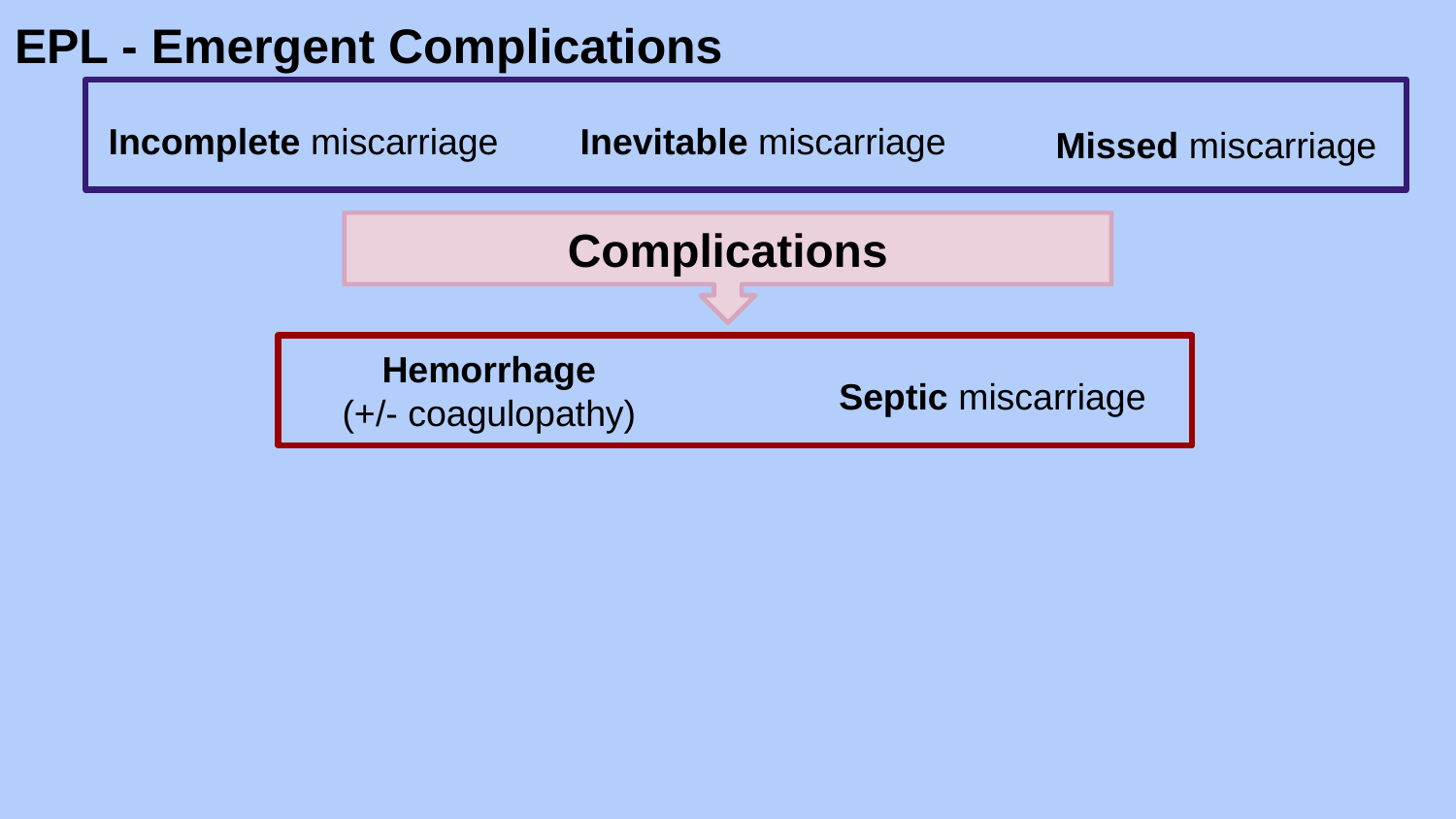

# EPL - Emergent Complications
Incomplete miscarriage
Inevitable miscarriage
Missed miscarriage
Complications
Hemorrhage
(+/- coagulopathy)
Septic miscarriage

## Slide 9
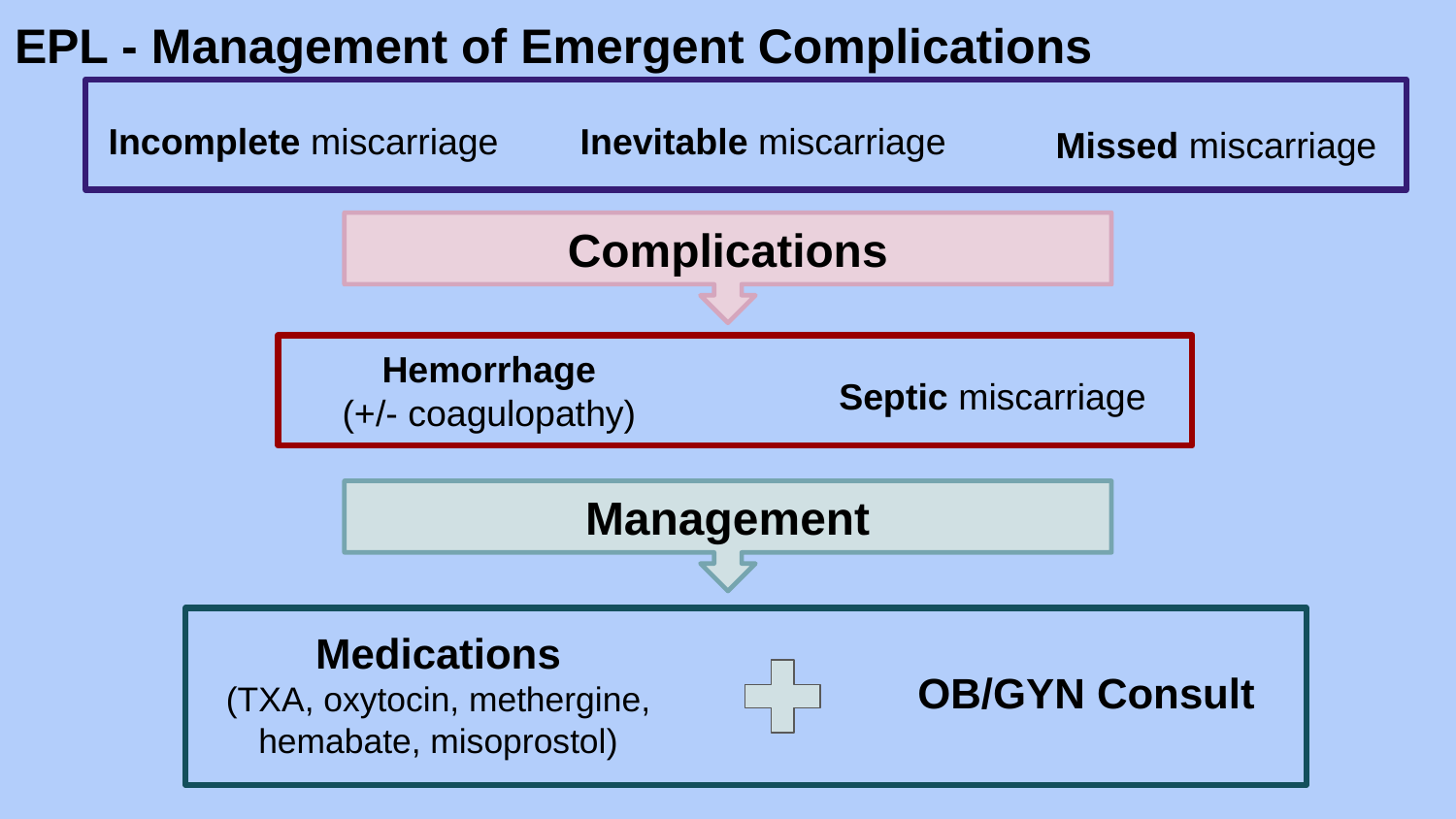

# EPL - Management of Emergent Complications
Incomplete miscarriage
Inevitable miscarriage
Missed miscarriage
Complications
Hemorrhage
(+/- coagulopathy)
Septic miscarriage
Management
Medications
(TXA, oxytocin, methergine, hemabate, misoprostol)
OB/GYN Consult

## Slide 10
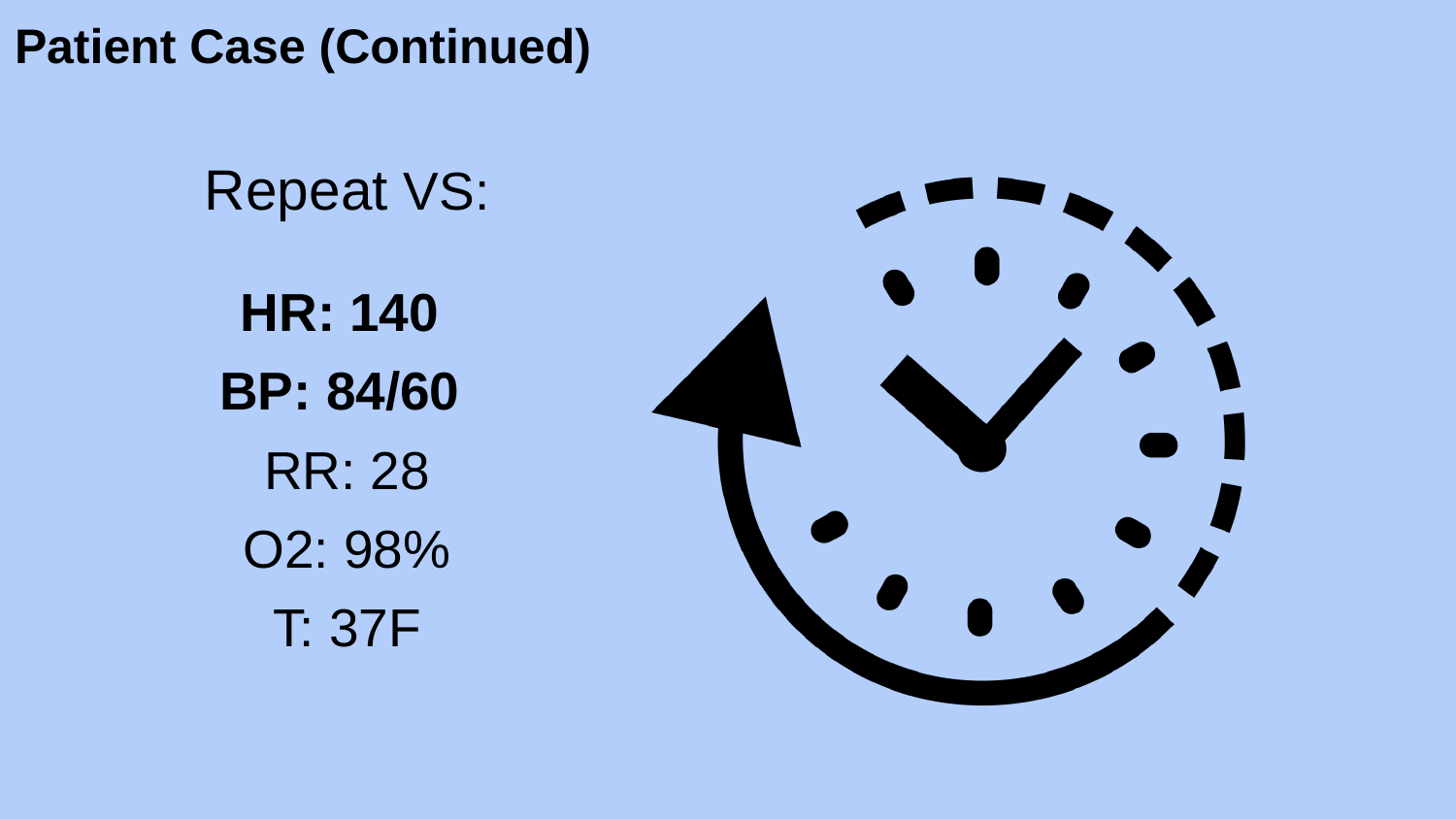

# Patient Case (Continued)
Repeat VS:
HR: 140
BP: 84/60
RR: 28
O2: 98%
T: 37F

## Slide 11
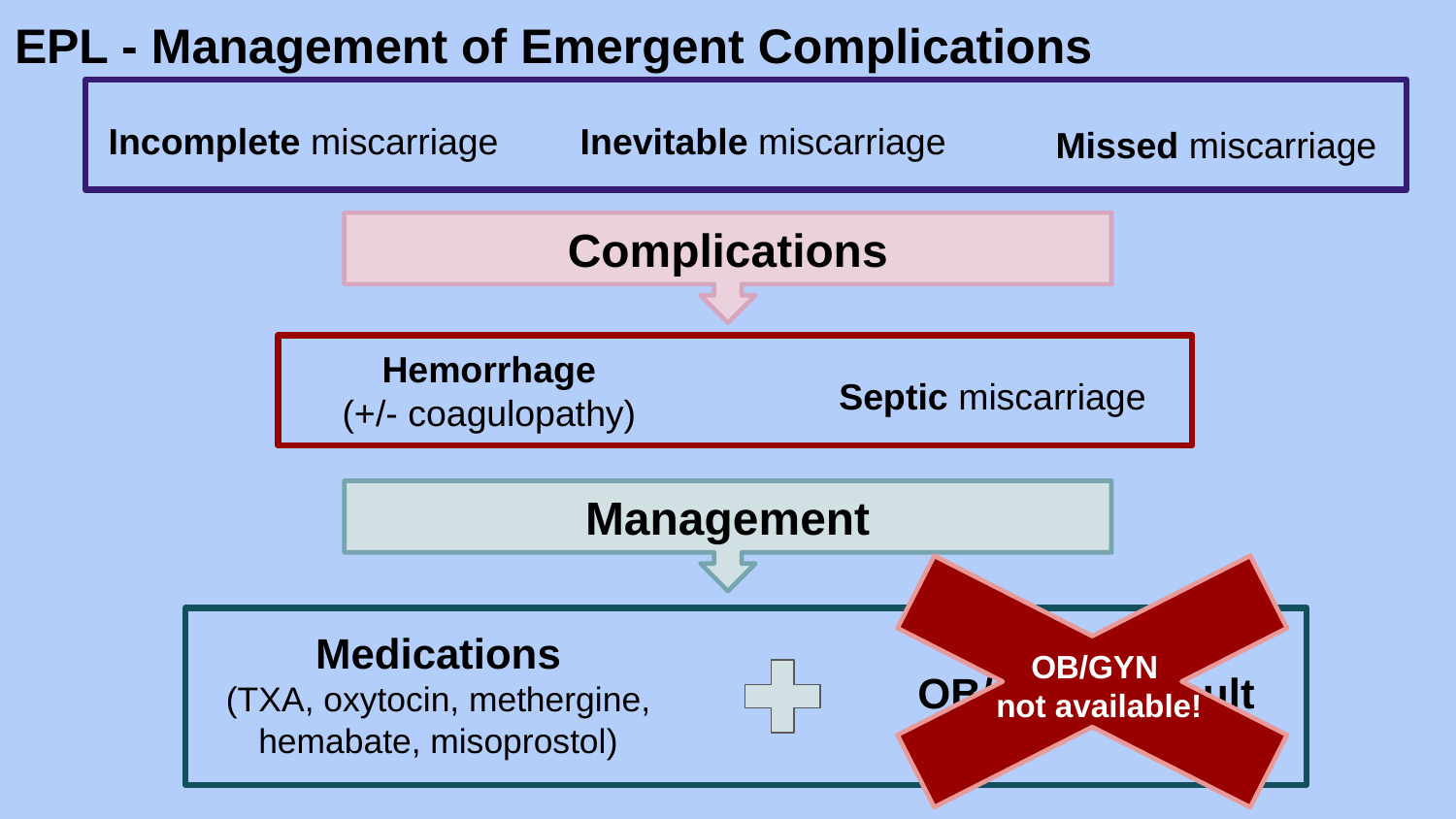

# EPL - Management of Emergent Complications
Incomplete miscarriage
Inevitable miscarriage
Missed miscarriage
Complications
Hemorrhage
(+/- coagulopathy)
Septic miscarriage
Management
Medications
(TXA, oxytocin, methergine, hemabate, misoprostol)
OB/GYN
not available!
OB/GYN Consult

## Slide 12
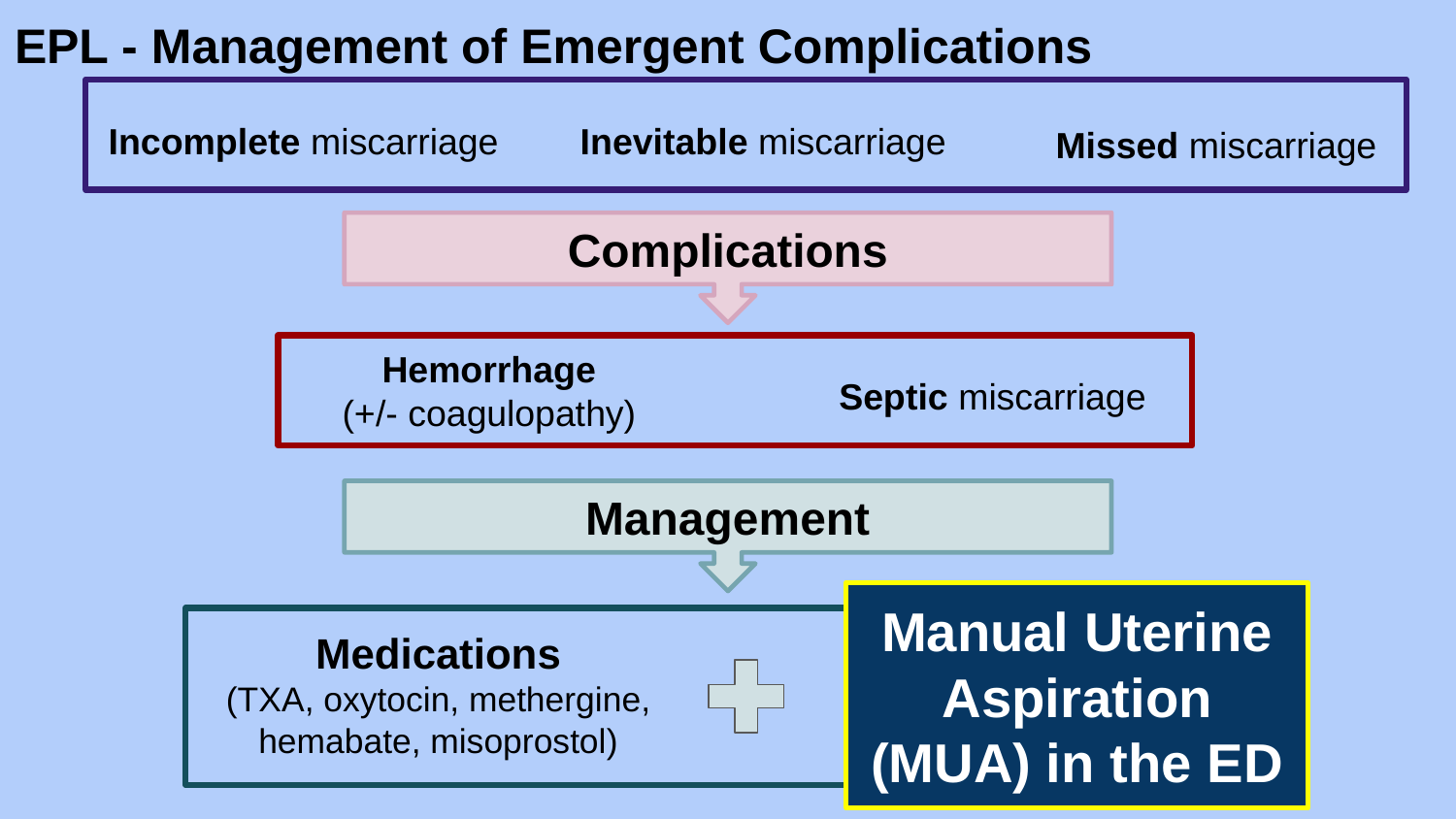

# EPL - Management of Emergent Complications
Incomplete miscarriage
Inevitable miscarriage
Missed miscarriage
Complications
Hemorrhage
(+/- coagulopathy)
Septic miscarriage
Management
Manual Uterine Aspiration (MUA) in the ED
Medications
(TXA, oxytocin, methergine, hemabate, misoprostol)

## Slide 13
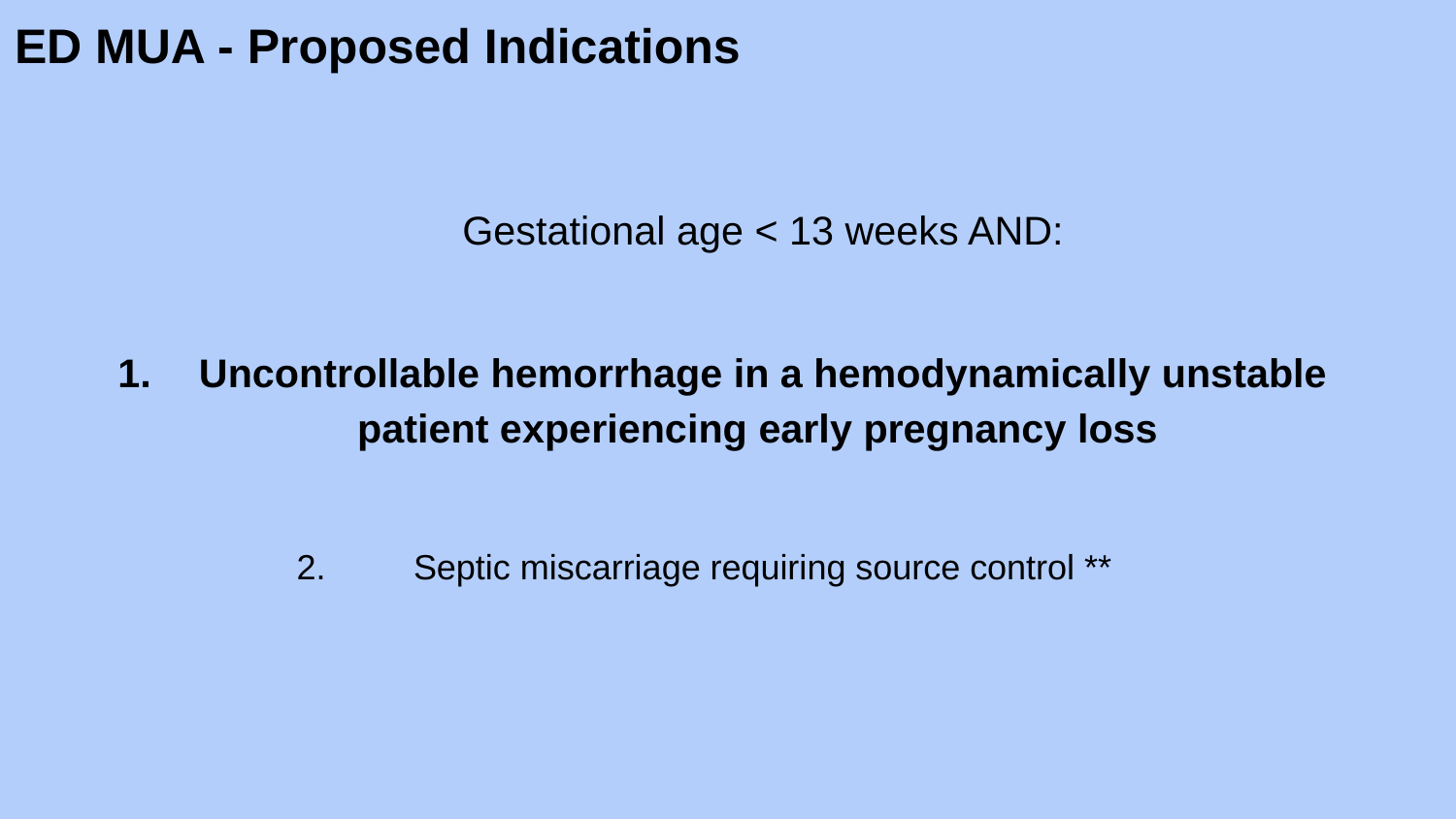

# ED MUA - Proposed Indications
Gestational age < 13 weeks AND:
Uncontrollable hemorrhage in a hemodynamically unstable patient experiencing early pregnancy loss
Septic miscarriage requiring source control **

## Slide 14
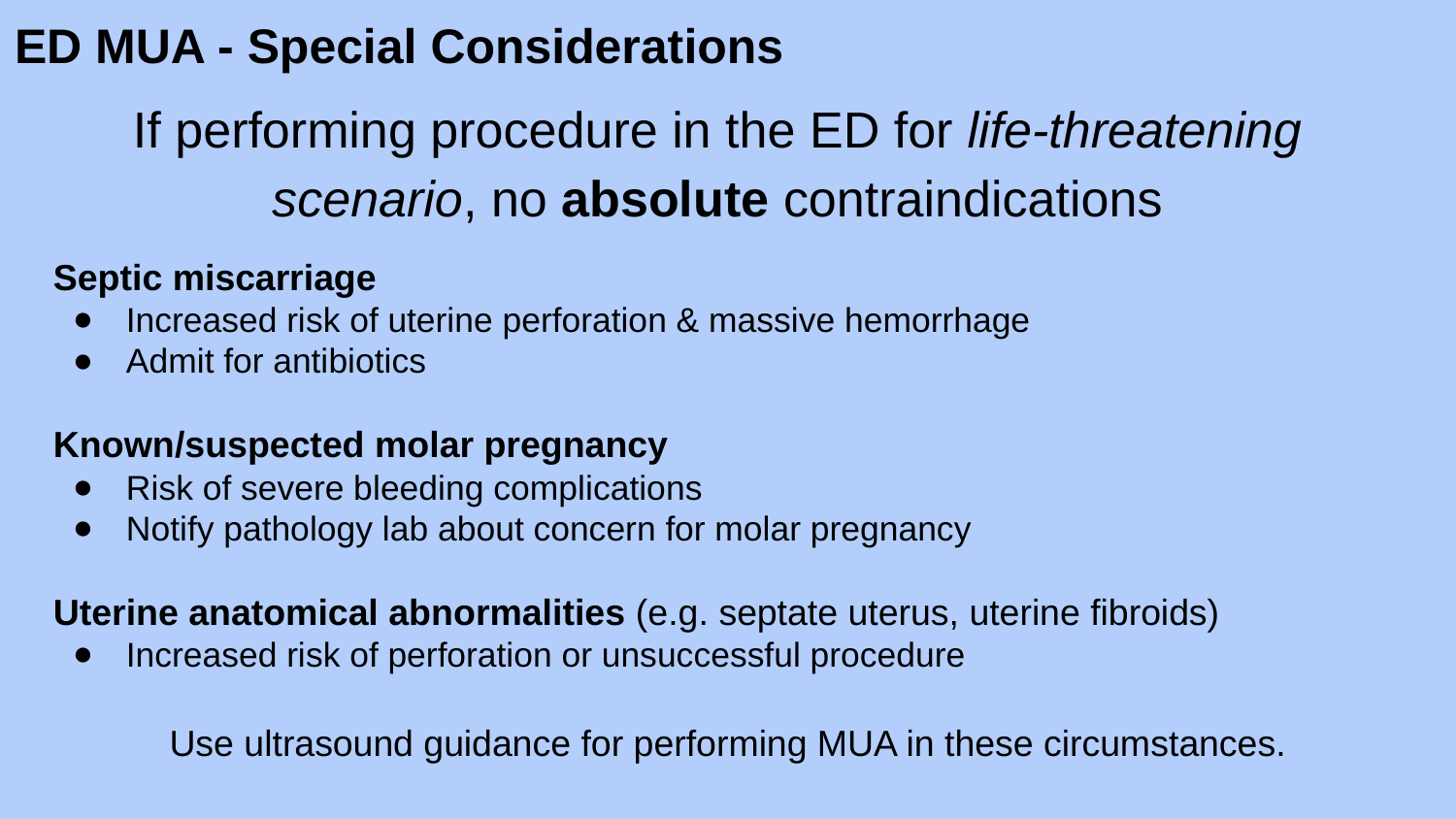

# ED MUA - Special Considerations
If performing procedure in the ED for life-threatening scenario, no absolute contraindications
Septic miscarriage
Increased risk of uterine perforation & massive hemorrhage
Admit for antibiotics
Known/suspected molar pregnancy
Risk of severe bleeding complications
Notify pathology lab about concern for molar pregnancy
Uterine anatomical abnormalities (e.g. septate uterus, uterine fibroids)
Increased risk of perforation or unsuccessful procedure
Use ultrasound guidance for performing MUA in these circumstances.

## Slide 15
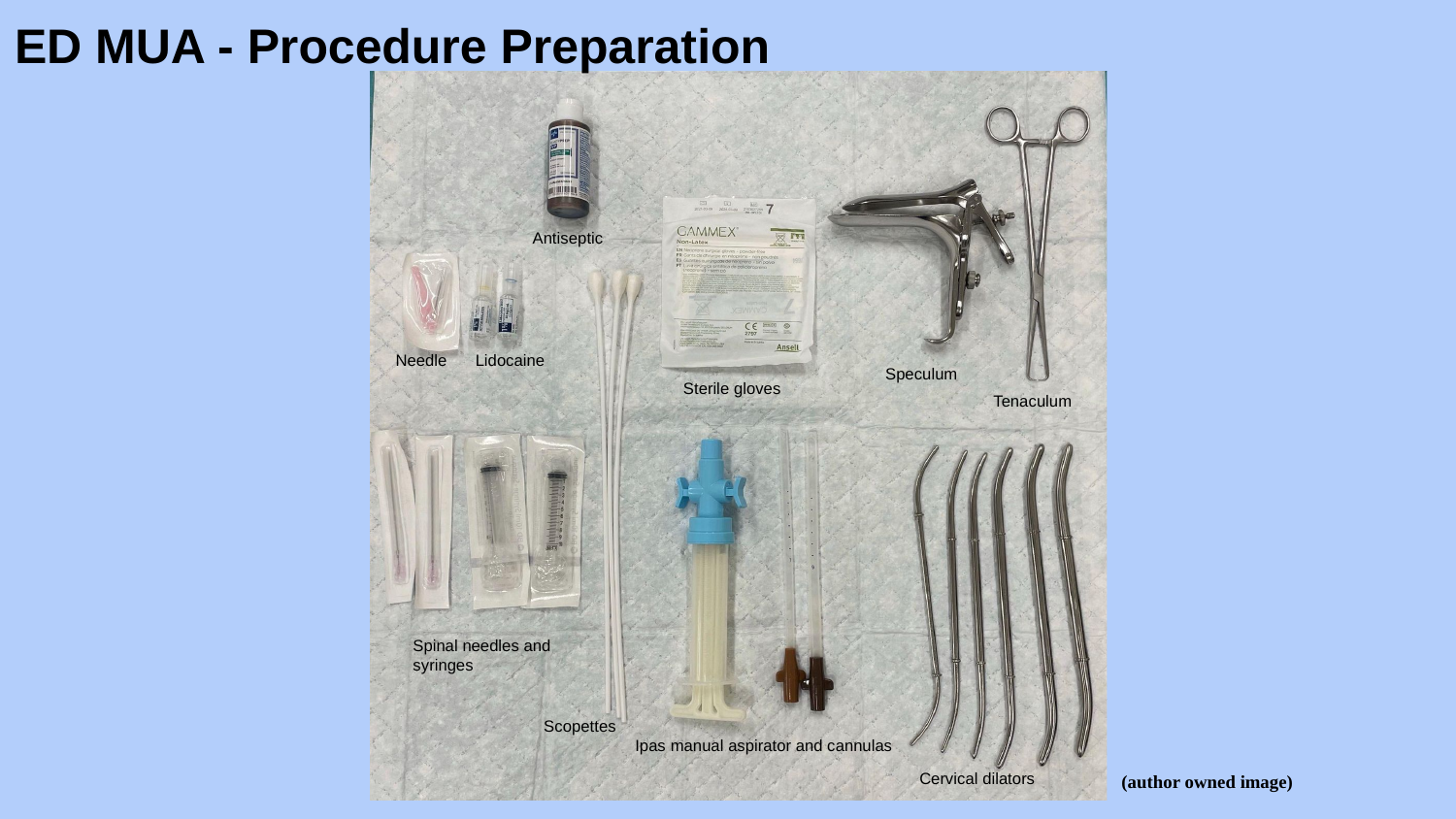

# ED MUA - Procedure Preparation
Antiseptic
Needle
Lidocaine
Speculum
Sterile gloves
Tenaculum
Spinal needles and syringes
Scopettes
Ipas manual aspirator and cannulas
Cervical dilators
(author owned image)

## Slide 16
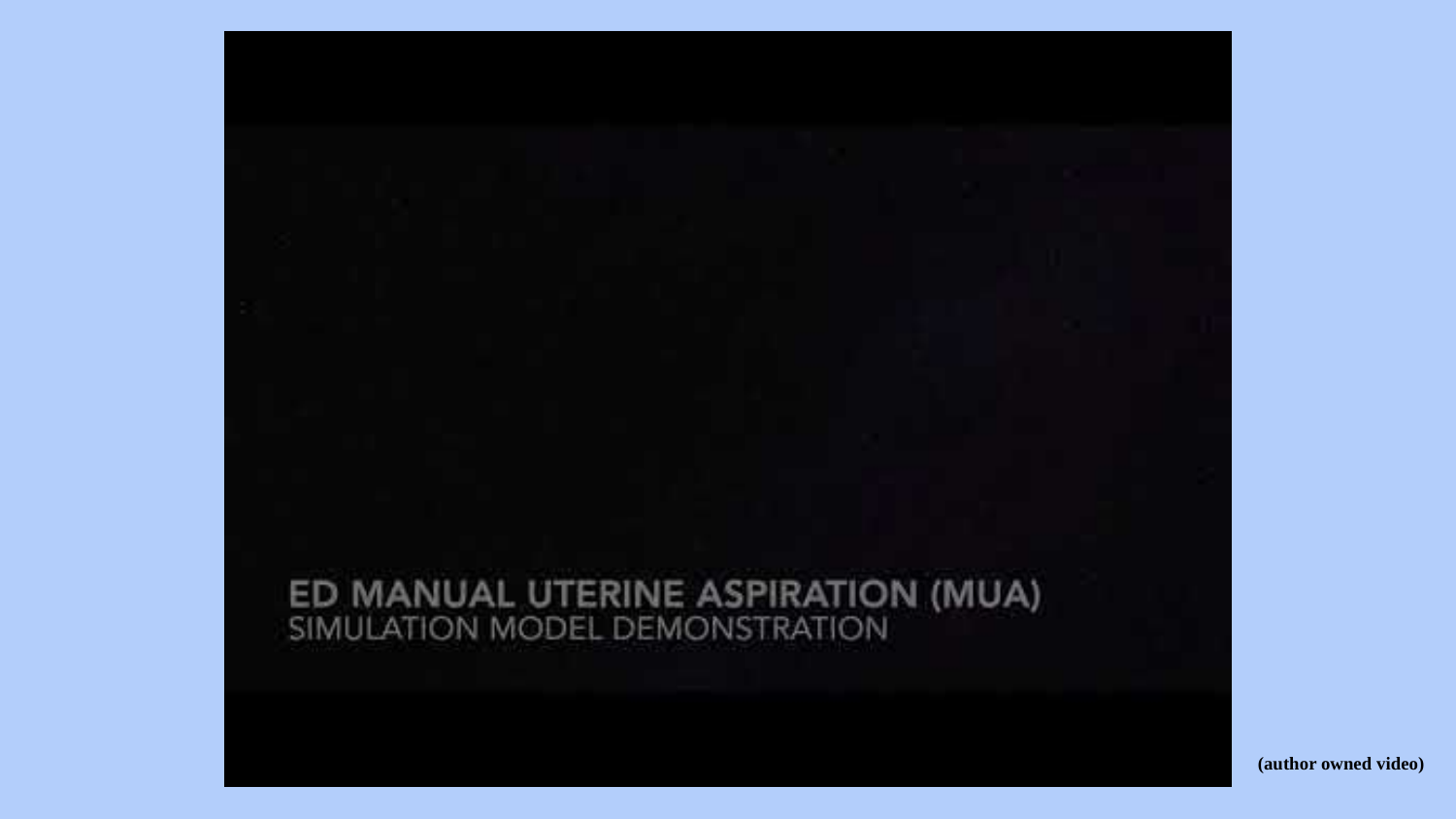

(author owned video)

## Slide 17
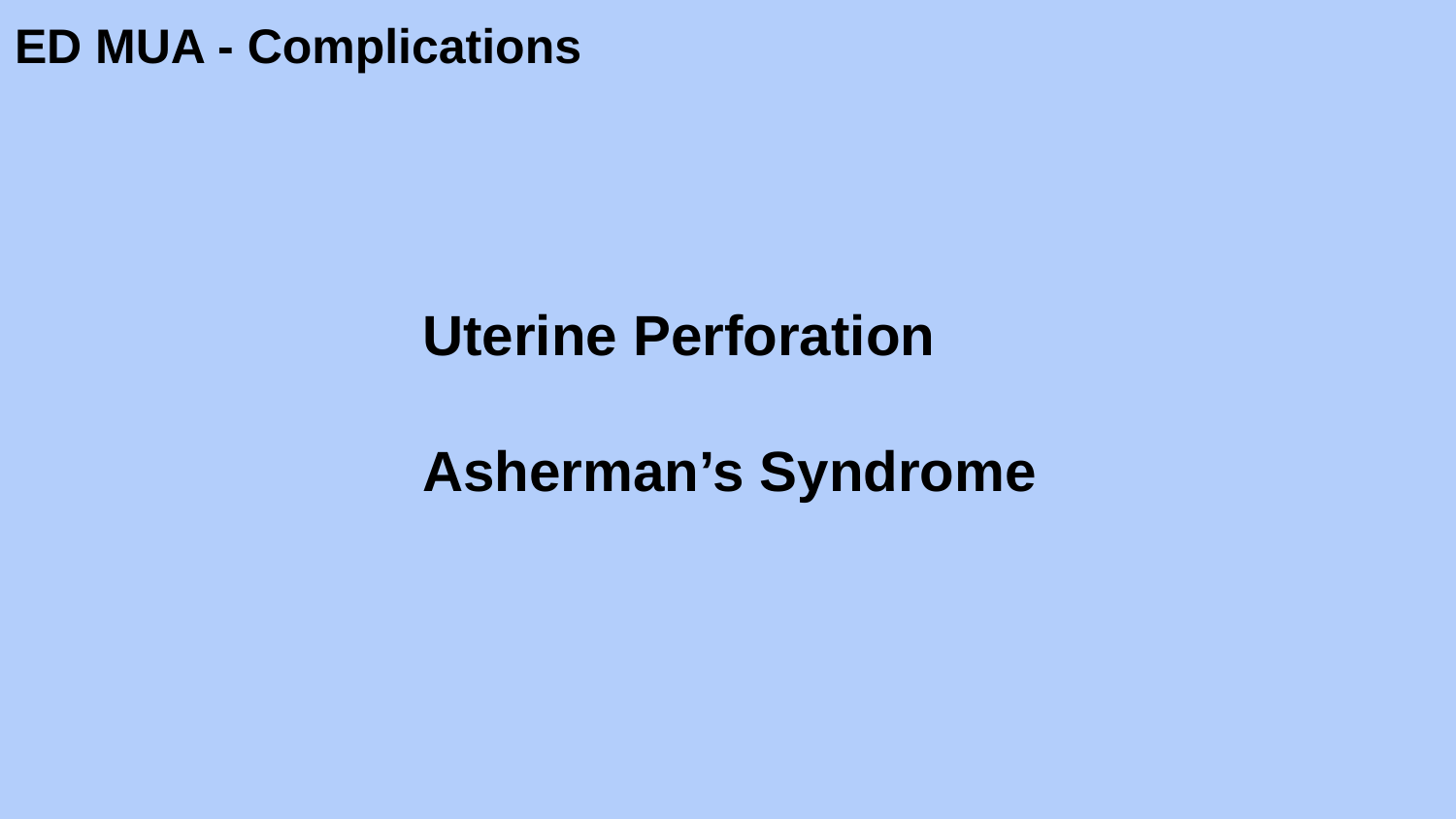

# ED MUA - Complications
Uterine Perforation
Asherman’s Syndrome

## Slide 18
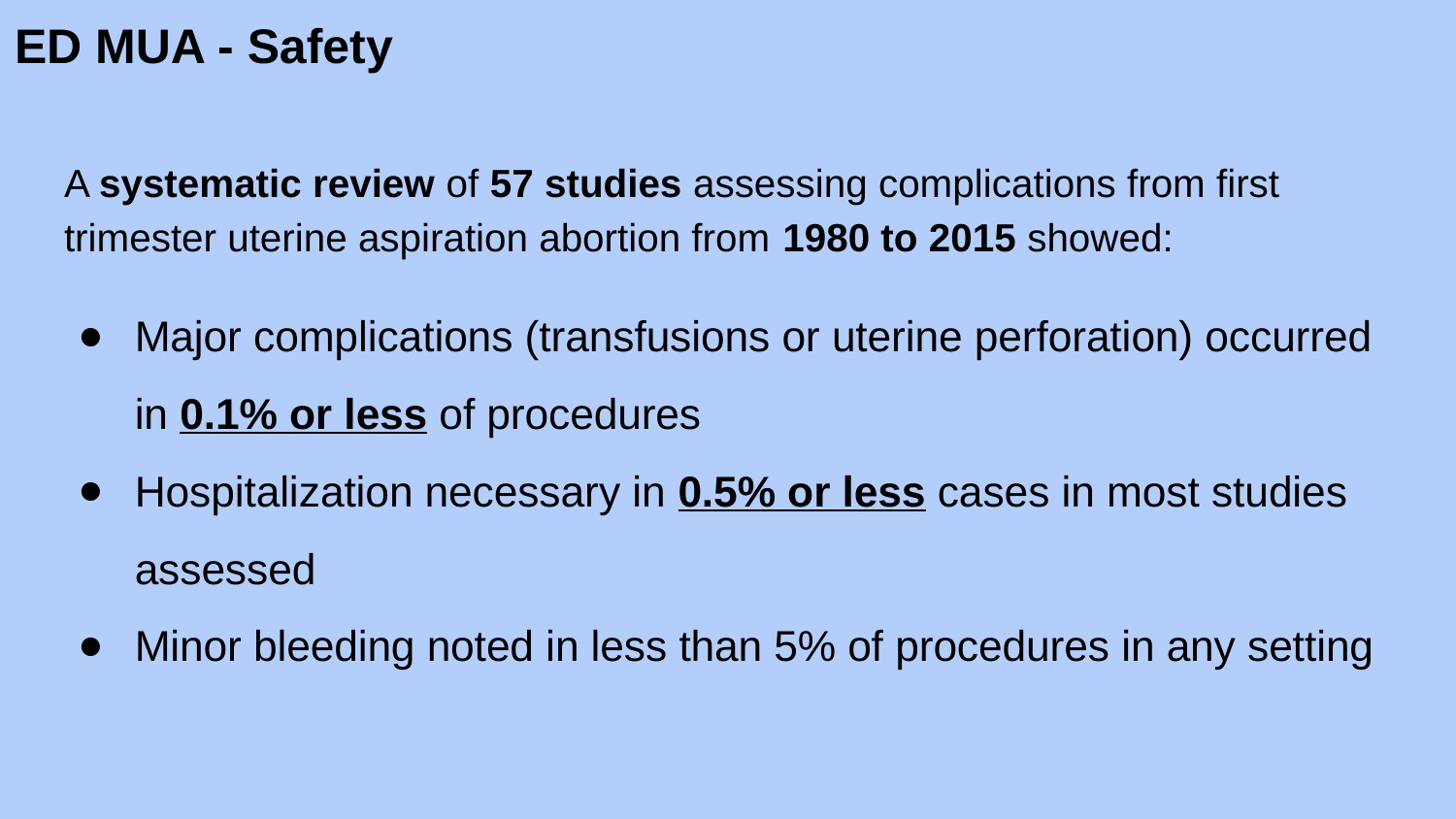

# ED MUA - Safety
A systematic review of 57 studies assessing complications from first trimester uterine aspiration abortion from 1980 to 2015 showed:
Major complications (transfusions or uterine perforation) occurred in 0.1% or less of procedures
Hospitalization necessary in 0.5% or less cases in most studies assessed
Minor bleeding noted in less than 5% of procedures in any setting

## Slide 19
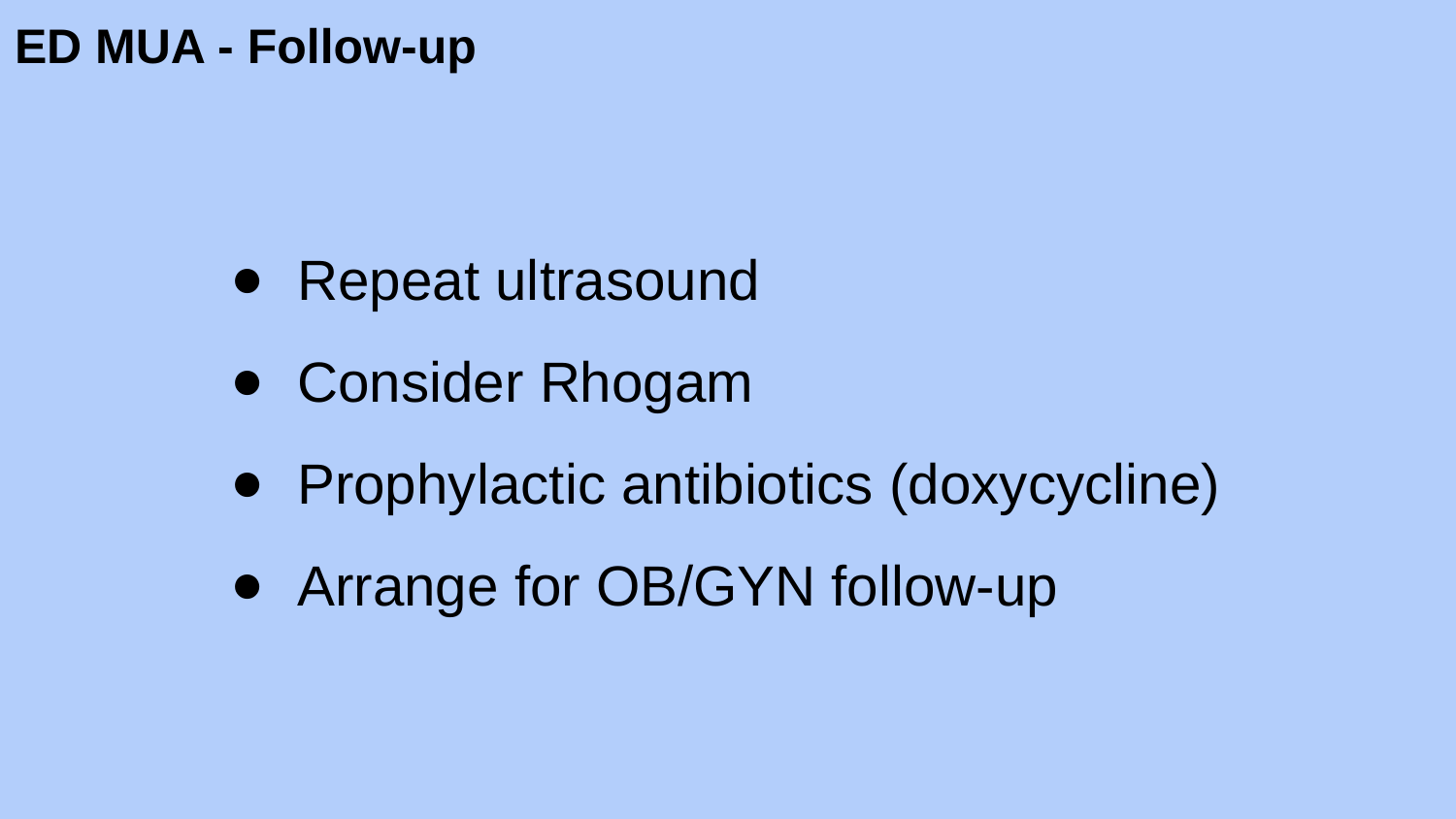

# ED MUA - Follow-up
Repeat ultrasound
Consider Rhogam
Prophylactic antibiotics (doxycycline)
Arrange for OB/GYN follow-up

## Slide 20
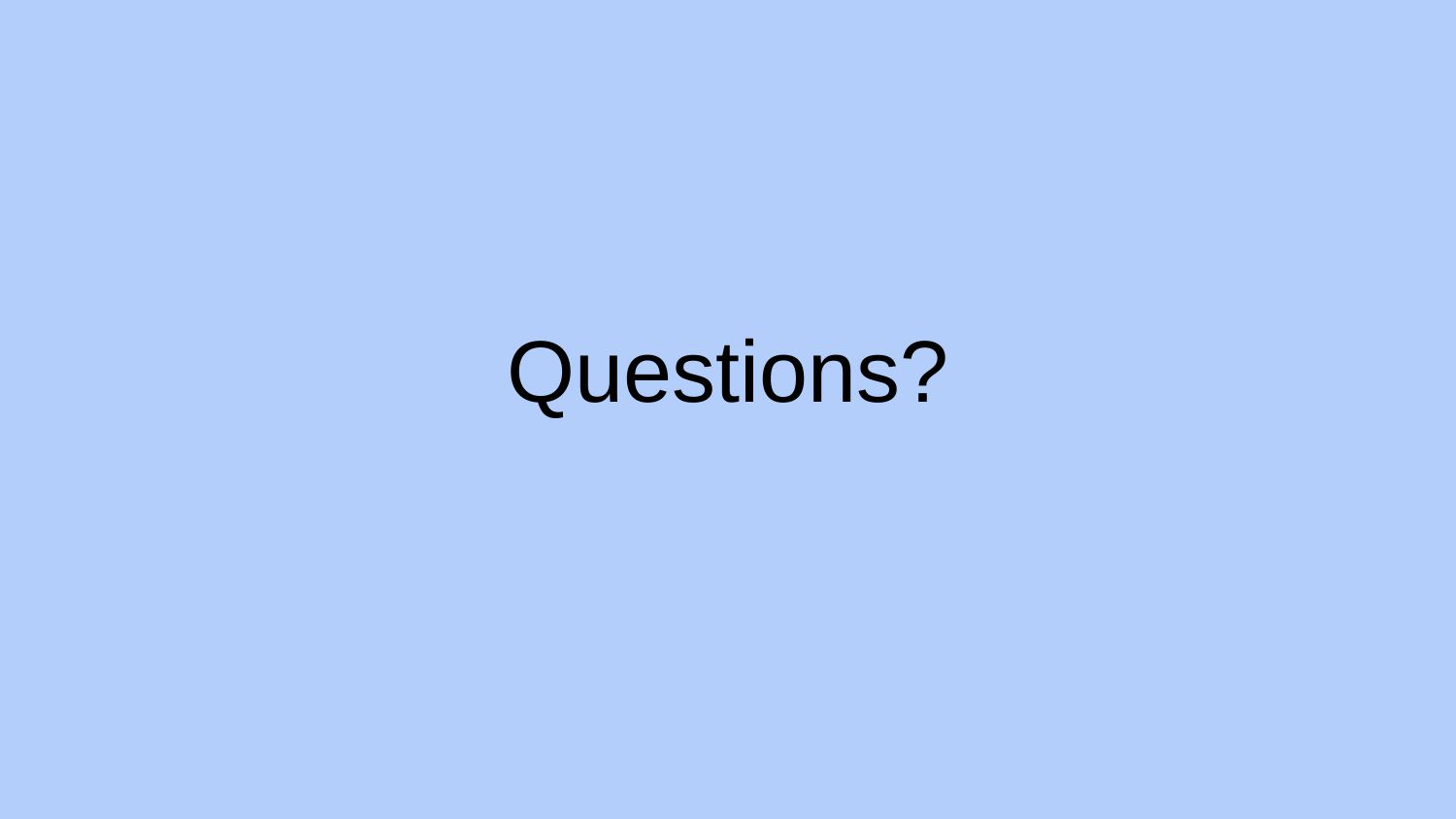

Questions?

## Slide 21
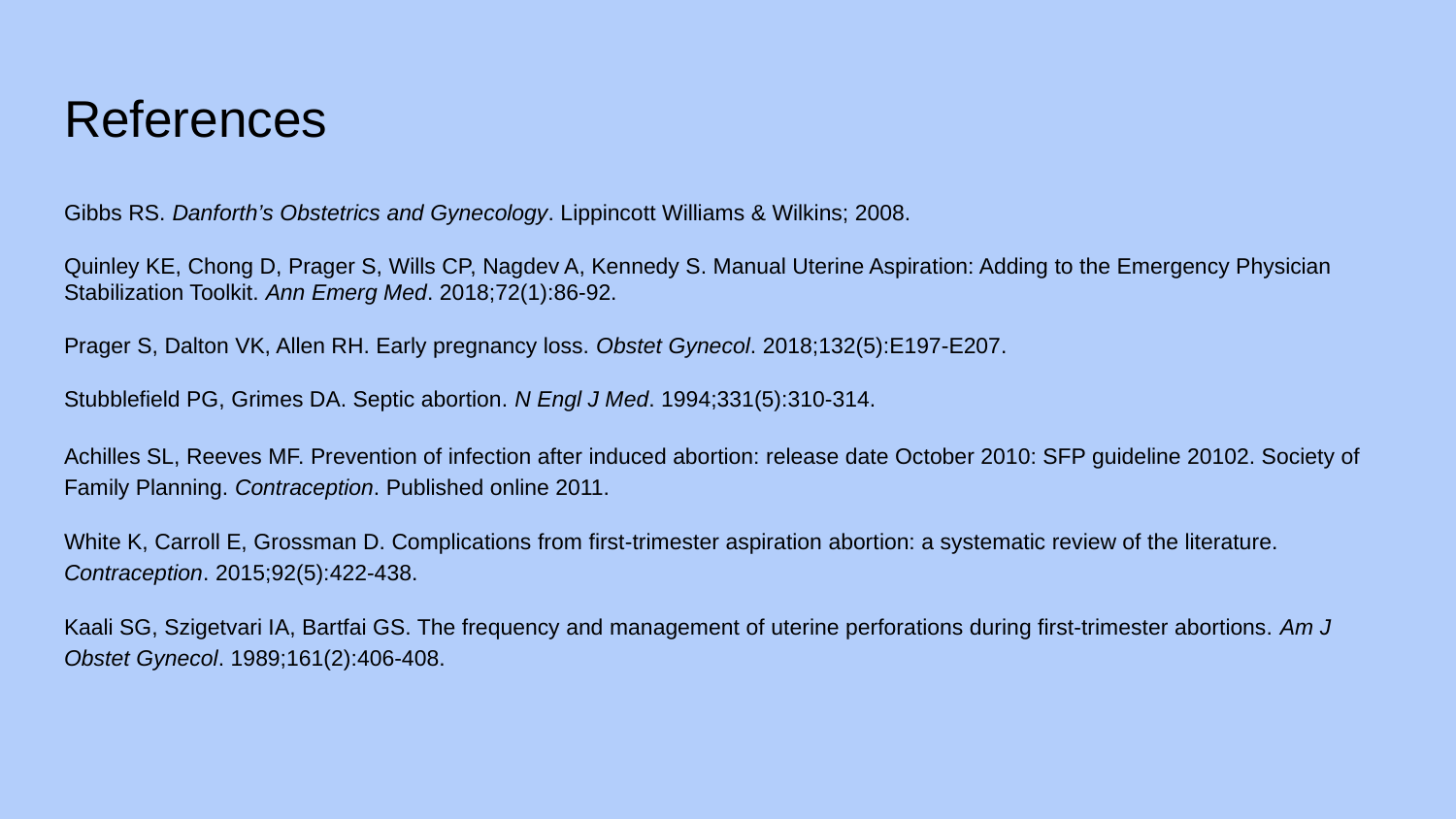

# References
Gibbs RS. Danforth’s Obstetrics and Gynecology. Lippincott Williams & Wilkins; 2008.
Quinley KE, Chong D, Prager S, Wills CP, Nagdev A, Kennedy S. Manual Uterine Aspiration: Adding to the Emergency Physician Stabilization Toolkit. Ann Emerg Med. 2018;72(1):86-92.
Prager S, Dalton VK, Allen RH. Early pregnancy loss. Obstet Gynecol. 2018;132(5):E197-E207.
Stubblefield PG, Grimes DA. Septic abortion. N Engl J Med. 1994;331(5):310-314.
Achilles SL, Reeves MF. Prevention of infection after induced abortion: release date October 2010: SFP guideline 20102. Society of Family Planning. Contraception. Published online 2011.
White K, Carroll E, Grossman D. Complications from first-trimester aspiration abortion: a systematic review of the literature. Contraception. 2015;92(5):422-438.
Kaali SG, Szigetvari IA, Bartfai GS. The frequency and management of uterine perforations during first-trimester abortions. Am J Obstet Gynecol. 1989;161(2):406-408.
