# Supplementary material for: Manual Uterine Aspiration Simulation for Emergency Medicine Learners
Source: MedEdPORTAL. 2024 Nov 11;20:11469. doi: 10.15766/mep_2374-8265.11469 (PMC11551269; doi:10.15766/mep_2374-8265.11469)
Supplement: Supplementary file 1 — MUA Model Preparation.docxStation Setup and Supplies.docxMUA Lecture.pptxMUA Video Demonstration.m4vFacilitator Guides.docxProcedure Checklist.docxLearner Survey.docxFacilitator Survey.docx [file mep_2374-8265.11469-s001.zip › E. Facilitator Guides.docx]

**Facilitator Guide 1: Cervical and Paracervical Anesthesia**

How to use this appendix: Provide this appendix at the start of the simulation for OB/Gyn facilitators to help them guide learners through the MUA stations. These guides will help the facilitators ensure every learner completes each station successfully and provide some helpful points for possible re-direction, if necessary, for simulation completion. The number of copies should equal the number of facilitators. Each station has a guide: cervical anesthesia, dilation, and complete MUA procedure.

This micro-skills session will utilize the simulated uterus outside of the pelvic model. At this station, the learner should proceed through local cervical anesthetic for tenaculum placement, placement of the tenaculum, and performance of the paracervical block.

1. Inform the learner that they are to assume that they have inserted the speculum, identified the cervix, and cleaned the cervix with an antiseptic. They should proceed from this step.
2. Inject 2 cc of lidocaine at 12 o’clock intracervical position. Emphasize that this is a local injection to provide anesthesia for the site of the tenaculum and is not a regional block.
   1. Ask the learner where they are injecting the lidocaine
      - 12 o’clock position into cervical tissue
   2. Ask how many cc’s of lidocaine they intend to inject at this location
      - Approximately 2 cc
3. Grip the cervix with a tenaculum.
   1. Ask the learner to demonstrate how they will then manipulate the tenaculum with their non-dominant hand.
   2. Ask where on the cervix they will grasp.
   3. Emphasize the hand positioning, allowing for manipulation of the tenaculum with their index finger and thumb while the 3rd-5th fingers stabilize against the bed or speculum.
4. Perform paracervical block.
   1. Emphasize that this is meant as a regional block to provide anesthesia for the entire cervix to facilitate cervical dilation.
   2. Inject 8 cc each at 4 and 8 o’clock paracervical position.
      - Ask the learner to describe the anatomic location where they will inject.
        - The cervicovaginal junction; where the vaginal fold meets the cervix.
      - Ask the learner what positions on the clock they will inject into at the cervicovaginal junction.
        - At 4 and 8 o’clock as this is close to the nerve supply to the cervix to allow for a regional block.
      - Ask which positions in the cervicovaginal junction they would particularly avoid and why.
        - 3 and 9 o’clock as this is where vasculature is more likely to course.

**Facilitator Guide 2: Cervical Dilation**

This microskills session will be done utilizing the simulated uterus outside of the pelvic model. Inform learners that they are to assume this is being performed with a speculum in place and that they have already antiseptically cleaned the cervix, the tenaculum is in place, and they have performed a paracervical block. At this station, the learner should perform serial cervical dilations. The focus is on becoming comfortable with holding the dilators and the level of pressure needed to dilate the cervix.

*Remind the learner that sterile technique/no touch is utilized for all of these steps*

1. Cervical Dilation
   1. Gentle traction on the tenaculum
   2. Serial dilation
      - Review the dilators being used and sizing of dilators
      - Ask the learner where they will hold the dilator, emphasizing the use of a pencil grip
      - Review rotating of dilator while maintaining sterility
      - Encourage steady gentle pressure with pass of first dilator

**Facilitator Guide 3: Complete MUA Procedure**

This final station is to allow the learner to perform a manual uterine aspiration from start to finish utilizing the microskills they have already practiced. For this station, the uterine model will be placed inside a pelvic model. The facilitator may choose to keep one hand on the uterine model to maintain its position in the pelvic model and provide counter pressure during the procedure. Learners should perform the entire procedure from the initial insertion of the speculum through aspiration and inspection of the cervix for post-procedural injuries.

1. Ask the learner to reiterate the indications and special considerations for this procedure.
2. Use of speculum to identify cervix.
3. Antiseptic cleaning of cervix.
4. Inject 2 cc lidocaine at 12 o’clock intracervical position.
   1. Ask the learner where anatomically they are injecting the lidocaine.
      - At the 12 o’clock position into cervical tissue.
   2. Ask how many cc’s of lidocaine they intend to inject at this location.
      - Approximately 2 cc.
5. Grip cervix with tenaculum.
   1. Ask the learner to demonstrate how they will then manipulate the tenaculum with their nondominant hand.
   2. Ask the learner where on the cervix they are placing the tenaculum.
6. Perform paracervical block.
   1. Inject approximately 8 cc at 4 o’clock position, switch syringes, and inject 10 cc at 8 o’clock position.
      - Ask the learner to describe the anatomic location they are injecting into.
        - The cervicovaginal junction; where the vaginal fold meets the cervix.
      - Ask the learner what positions on the clock they will inject into.
        - The 4 and 8 o’clock positions.
      - Ask which positions they would like to avoid.
        - The 3 and 9 o’clock as this is where vasculature is more likely to course.

*Remind the learner that sterile technique is utilized for each of these steps*

1. Cervical dilation
   1. Gentle traction on tenaculum
   2. Serial dilation
      - Ask the learning where they will hold the dilator.
        - Center of the dilator (pencil grip) to avoid contaminating the ends.
2. Prepare aspirator
   1. Match cannula size to the week of gestation or one size smaller.
   2. Press two tabs at the proximal aspect of the aspirator to prime the suction.
   3. Withdraw aspirator plunger to create suction.
3. Uterine aspiration
   1. Remind the learner to apply steady constant pressure for insertion.
      - Ask the learner what they should do if they believe their cannula is abutting the uterine fundus.
        - Withdraw 2cm.
   2. Release aspirator vacuum.
   3. Slowly remove the aspirator utilizing 360-degree rotatory movement of the cannula.
      - Prompt learner to withdraw cannula with 360-degree rotatory movements, if not initially done.
   4. Identify gritty texture/sound when complete.
4. Procedure completion
   1. Remove aspirator.
   2. Remove tenaculum.
   3. Examine cervix.
      - Remind the learner to perform a final inspection of the cervix for damage prior to removing speculum.
