# Supplementary material for: Manual Uterine Aspiration Simulation for Emergency Medicine Learners
Source: MedEdPORTAL. 2024 Nov 11;20:11469. doi: 10.15766/mep_2374-8265.11469 (PMC11551269; doi:10.15766/mep_2374-8265.11469)
Supplement: Supplementary file 1 — MUA Model Preparation.docxStation Setup and Supplies.docxMUA Lecture.pptxMUA Video Demonstration.m4vFacilitator Guides.docxProcedure Checklist.docxLearner Survey.docxFacilitator Survey.docx [file mep_2374-8265.11469-s001.zip › F. Procedure Checklist.docx]

**Checklist for Manual Uterine Aspiration:**

How to use this appendix: Provide this for the facilitators at the start of the training to use during the simulation to ensure each learning successfully performs all steps of MUA for simulation completion. The number of copies should equal the number of learners.

*Please check these off as the learner completes each portion of the procedure.*

1. Ask the learner to name the indications for MUA and special _____

considerations that make it a higher risk.

1. Use of speculum to identify cervix _____
2. Antiseptic cleaning of cervix _____
3. Inject local anesthetic at 12 o’clock intracervical position _____
   1. Describe the anatomic location they are injecting
   2. Describe what positions they would like to avoid
4. Grip cervix with tenaculum _____
5. Paracervical block _____
   1. Inject local anesthetic at the 4 o’clock and 8 o’clock positions
6. Switch to sterile technique _____
7. Cervical dilation _____
   1. Apply gentle traction on tenaculum
   2. Perform serial dilation
8. Prepare uterine aspirator _____
   1. Attach cannula to aspirator
   2. Create suction within aspirator
9. Uterine aspiration _____
   1. Insert aspirator through cervical canal with steady pressure
   2. Release aspirator vacuum
   3. Withdraw cannula using a 360 degree rotatory movement

1. Aspiration completion _____
   1. Recognize that no additional POC being withdrawn

on aspiration attempts

- 1. Identify gritty texture/sound

1. Remove aspirator and speculum _____
   1. Examine cervix for injury
