# Supplementary material for: Manual Uterine Aspiration Simulation for Emergency Medicine Learners
Source: MedEdPORTAL. 2024 Nov 11;20:11469. doi: 10.15766/mep_2374-8265.11469 (PMC11551269; doi:10.15766/mep_2374-8265.11469)
Supplement: Supplementary file 1 — MUA Model Preparation.docxStation Setup and Supplies.docxMUA Lecture.pptxMUA Video Demonstration.m4vFacilitator Guides.docxProcedure Checklist.docxLearner Survey.docxFacilitator Survey.docx [file mep_2374-8265.11469-s001.zip › G. Learner Survey.docx]

**Participant MUA Curriculum Evaluation**

How to use this appendix: Provide this self-evaluation to all learners immediately upon completing the simulation training as part of the curricular evaluation. Collect the anonymous responses at the end. The number of copies available should equal the number of learners.

Please complete this self-assessment about your feelings and knowledge before and after participating in this novel simulation curriculum on emergency department manual uterine aspiration (MUA).

Honest responses will help us evaluate whether this simulation curriculum is effective and worthwhile.

These responses will be kept anonymous and will not be tied to your identity as a participant.

Rate your **level of confidence** in the following domains **PRIOR TO** the educational session and **NOW.**

**How confident are you in your**

|  | PRIOR TO THE SESSION | | | | | NOW | | | | |
| --- | --- | --- | --- | --- | --- | --- | --- | --- | --- | --- |
|  | Not at all confident | Slightly Confident | Moderately Confident | Quite Confident | Extremely Confident | Not at all confident | Slightly Confident | Moderately Confident | Quite Confident | Extremely Confident |
| Ability to identify indications to performing an ED MUA | ☐ | ☐ | ☐ | ☐ | ☐ | ☐ | ☐ | ☐ | ☐ | ☐ |
| Ability to identify higher risk clinical scenarios in performing an ED MUA | ☐ | ☐ | ☐ | ☐ | ☐ | ☐ | ☐ | ☐ | ☐ | ☐ |

Rate your **level of comfort** in the following domains **PRIOR TO** the educational session and **NOW**

**How comfortable are you in your ability to**

|  | PRIOR TO THE SESSION | | | | | NOW | | | | |
| --- | --- | --- | --- | --- | --- | --- | --- | --- | --- | --- |
|  | **Very Uncomfortable** | **Uncomfortable** | **Neutral** | **Comfortable** | **Very Comfortable** | **Very Uncomfortable** | **Uncomfortable** | **Neutral** | **Comfortable** | **Very Comfortable** |
| Perform a paracervical block | **☐** | **☐** | **☐** | **☐** | **☐** | **☐** | **☐** | **☐** | **☐** | **☐** |
| Perform cervical dilation | **☐** | **☐** | **☐** | **☐** | **☐** | **☐** | **☐** | **☐** | **☐** | **☐** |
| Aspirate uterine contents | **☐** | **☐** | **☐** | **☐** | **☐** | **☐** | **☐** | **☐** | **☐** | **☐** |
| Perform an MUA from start to finish | **☐** | **☐** | **☐** | **☐** | **☐** | **☐** | **☐** | **☐** | **☐** | **☐** |

Based on this curriculum, in the appropriate clinical context, how likely are you to consider performing MUA as management for life-threatening complications of EPL?

| Very Unlikely | Unlikely | Neutral | Likely | Very Likely |
| --- | --- | --- | --- | --- |
| ☐ | ☐ | ☐ | ☐ | ☐ |

**Please evaluate this session as a learning experience**.

After this session, how interested are you in further education/experience with MUA in the emergency setting?

| Very Uninterested | Uninterested | Neutral | Interested | Very Interested |
| --- | --- | --- | --- | --- |
| ☐ | ☐ | ☐ | ☐ | ☐ |

How effective was this curriculum overall?

| Not at all Effective | Somewhat Effective | Moderately Effective | Quite Effective | Extremely Effective |
| --- | --- | --- | --- | --- |
| ☐ | ☐ | ☐ | ☐ | ☐ |

## How effective was the video demonstration?

| Not at all Effective | Somewhat Effective | Moderately Effective | Quite Effective | Extremely Effective |
| --- | --- | --- | --- | --- |
| ☐ | ☐ | ☐ | ☐ | ☐ |

## How effective were the facilitators?

| Not at all Effective | Somewhat Effective | Moderately Effective | Quite Effective | Extremely Effective |
| --- | --- | --- | --- | --- |
| ☐ | ☐ | ☐ | ☐ | ☐ |

## How effective was the simulation model?

| Not at all Effective | Somewhat Effective | Moderately Effective | Quite Effective | Extremely Effective |
| --- | --- | --- | --- | --- |
| ☐ | ☐ | ☐ | ☐ | ☐ |

What worked well in this session?

|  |
| --- |
|  |

What could be improved?

|  |
| --- |
|  |

Any additional feedback?

|  |
| --- |
|  |
