# Supplementary material for: Manual Uterine Aspiration Simulation for Emergency Medicine Learners
Source: MedEdPORTAL. 2024 Nov 11;20:11469. doi: 10.15766/mep_2374-8265.11469 (PMC11551269; doi:10.15766/mep_2374-8265.11469)
Supplement: Supplementary file 1 — MUA Model Preparation.docxStation Setup and Supplies.docxMUA Lecture.pptxMUA Video Demonstration.m4vFacilitator Guides.docxProcedure Checklist.docxLearner Survey.docxFacilitator Survey.docx [file mep_2374-8265.11469-s001.zip › H. Facilitator Survey.docx]

**Facilitator Evaluation of the Curriculum**

How to use this appendix: This survey should be distributed as part of the curricular evaluation after the workshop. The number of copies should equal the number of facilitators.

Thank you very much for being involved in today's simulation session. This was the first attempt at this skills session and your feedback would be very much appreciated to improve this session for future learners. We would appreciate it if you took a moment to answer the questions below.

How effective was this curriculum overall?

| Not at all Effective | Somewhat Effective | Moderately Effective | Quite Effective | Extremely Effective |
| --- | --- | --- | --- | --- |
| ☐ | ☐ | ☐ | ☐ | ☐ |

## How effective was the simulation model?

| Not at all Effective | Somewhat Effective | Moderately Effective | Quite Effective | Extremely Effective |
| --- | --- | --- | --- | --- |
| ☐ | ☐ | ☐ | ☐ | ☐ |

## How useful was the facilitator guide?

| Not at all useful | Somewhat useful | Moderately useful | Quite useful | Extremely useful |
| --- | --- | --- | --- | --- |
| ☐ | ☐ | ☐ | ☐ | ☐ |

What worked well in this session?

|  |
| --- |
|  |

What could be improved?

|  |
| --- |
|  |

Any additional feedback?

|  |
| --- |
|  |
