# Supplementary material for: Manual Uterine Aspiration Simulation for Emergency Medicine Learners
Source: MedEdPORTAL. 2024 Nov 11;20:11469. doi: 10.15766/mep_2374-8265.11469 (PMC11551269; doi:10.15766/mep_2374-8265.11469)
Supplement: Supplementary file 1 — MUA Model Preparation.docxStation Setup and Supplies.docxMUA Lecture.pptxMUA Video Demonstration.m4vFacilitator Guides.docxProcedure Checklist.docxLearner Survey.docxFacilitator Survey.docx [file mep_2374-8265.11469-s001.zip › _Educational Summary Report_11469.pdf]

MedEdPORTAL® The AAMC Journal of Teaching and Learning Resources

Original Publication Open Access

Manual Uterine Aspiration Simulation for Emergency Medicine Learners

Katherine Wegman, MD[†], Caroline Gorka, MD[†], Judith Linden, MD, Shannon Bell, MD, Stephanie N. Stapleton, MD, Virginia Tancioco, MD, Laura Welsh, MD[*,†]

*Corresponding author: lwelsh@mgb.org
[†]Co-primary author

Abstract

Introduction: Manual uterine aspiration is a potentially lifesaving procedure for treating patients with hemorrhagic complications of early pregnancy loss. While early pregnancy loss is a common diagnosis seen in the emergency department, manual uterine aspiration education is lacking for emergency medicine physicians. **Methods:** We designed a 90-minute procedural skills training session for 30 emergency medicine learners. The session included a brief lecture and video demonstration, followed by two micro-skills stations before finally completing the simulated procedure in its entirety. At each station, learners were asked to verbalize the steps and landmarks for the procedure before performing them on models. Participants completed a combined pre-post survey evaluating their perceived knowledge of the procedure and self-efficacy in performing the procedure. **Results:** Thirty learners who participated in the workshop were surveyed, with a 100% response rate. All participants reported increased comfort with the procedure and knowledge about the procedure. All participants completed a successful simulated procedure. Participants also indicated increased interest in learning more about manual uterine aspiration and its potential application within the emergency medicine physician's scope of practice. **Discussion:** We developed a workshop to train emergency medicine learners in manual uterine aspiration to stop life-threatening hemorrhage in the setting of early pregnancy loss. The workshop was well received by learners and increased their self-efficacy and desire for additional training with this procedure. Similar curricula should be tried at other institutions.

Keywords
Early Pregnancy Loss, Manual Uterine Aspiration, Women's Health, Clinical/Procedural Skills Training, Emergency Medicine, OB/GYN, Simulation

Educational Objectives

By the end of this session, participants should be able to:

1. Recognize indications for emergency department manual uterine aspiration (MUA) for the management of early pregnancy loss.
2. Explain special considerations where MUA would be considered a higher-risk procedure.
3. Identify the correct landmarks for a paracervical block.
4. Demonstrate the steps necessary to perform cervical dilation.
5. Perform a successful simulated MUA.
6. Express increased comfort in their ability to perform MUA for complications of early pregnancy loss.

Citation:
Wegman K, Gorka C, Linden J, et al. Manual uterine aspiration simulation for emergency medicine learners. *MedEdPORTAL*. 2024;20:11469. https://doi.org/10.15766/mep_2374-8265.11469

Introduction

Early pregnancy loss (EPL) is the most common complication of pregnancy, occurring in at least 10%-15% of clinically recognized pregnancies.[1] EPL is defined as a nonviable intrauterine pregnancy with either an empty gestational sac or a gestational sac containing a conceptus without fetal heart activity at less than 13 weeks of gestation.[1] EPL-related care accounts for an estimated 900,000 emergency department (ED) visits annually, and the ED is often the first health care system touchpoint for women experiencing vaginal bleeding as a complication of EPL.[2,3] Despite this, there is a paucity of evidence informing the ED management of critically ill patients with hemodynamic instability as a result of EPL.

Emergency medicine (EM) training for this high-acuity scenario typically focuses on medical management and temporizing measures for these patients. However, for hemodynamically unstable patients with EPL-related hemorrhage, prompt evacuation of the uterus is recommended for definitive

Copyright © 2024 Wegman et al. This is an open-access publication distributed under the terms of the Creative Commons Attribution license.

management.[1] This procedure is known as manual uterine aspiration (MUA). MUA is a safe, cost-effective procedure that has been effectively incorporated into emergency settings with improved patient care.[4] Additionally, interdisciplinary MUA training for hemorrhagic EPL facilitates a better understanding of this procedure by ED physicians and has been shown to improve the overall care of patients with EPL in the ED.[5]

Emergency physicians do not routinely receive formal training in this procedure despite its potential utility in the ED setting. MUA can be a critical procedure for EM physicians who practice in lower-resourced settings without ready access to gynecology specialists. MUA is an infrequent but potentially lifesaving procedure to have within the EM skill set—a skill set that already features several other rare but lifesaving procedures, including resuscitative hysterotomy and thoracotomy. There have been increasing calls to incorporate MUA into EM training and descriptions of its use in the ED.[6-8] However, at this time, we are unaware of any published curricula on MUA for EM physicians.

The goal of this curriculum was to improve EM learners' ability to definitively manage patients with hemorrhagic complications of EPL by introducing them to the concept of MUA and practicing the procedure. The curriculum was developed through the framework of deliberate practice and mastery learning. Deliberate practice allows for structured practice with directed, real-time feedback in order to achieve effective skill proficiency.[9,10] Mastery learning utilizes a stepwise learning approach of increasing difficulty with clear achievement standards.[11] Together, mastery learning and deliberate practice can improve procedural skills and increase professional self-efficacy.[10,11]

Methods

This training session was implemented as part of a regular monthly simulation session for EM learners within the residency curriculum. It consisted of an initial didactic component followed by a procedural simulation session. Participants included EM fourth-year clerkship students, PGY 1-PGY 4 EM residents, and pediatric EM fellows who were PGY 4-PGY 5, having previously completed a pediatric residency. None of the EM learners had prior formal training on the use of MUA in an emergency setting. Based on their level of training, we assumed our learners had prerequisite knowledge of procedural and examination skills including maintenance of sterile technique, speculum placement, and drawing up and injecting local anesthetics.

Curricular Development

This curriculum was designed by two board-certified EM physicians with specialized training in simulation and medical education, two board-certified obstetrics and gynecology physicians, and two senior EM residents. All the educational materials for the session, including slides, video, content, facilitator guides, and checklists, were reviewed and edited by members of the team, including the gynecology content experts.

Prior to the simulation session, uterine models were built, and the procedure was trialed on the models by three novice EM learners. The purpose of the trial session was twofold: first, to determine the approximate time allotments necessary for a novice EM learner to complete each step of the MUA procedure, and second, to evaluate the integrity of the models after repeated use. We determined through our trial session that each uterine model could be used two to three times before it needed to be replaced or repaired. To this end, we prepared a total of 24 uterine models for an anticipated 30-40 participants in our session, with eight extra models available in the event of unforeseen model malfunctions.

Equipment

For the didactic portion of the curriculum, a project system was needed for the introductory lecture and video. For the simulation section, we used a uterine MUA model originally designed by Dr. Meg O'Reilly from the Department of Obstetrics and Gynecology at the Oregon Health & Science University School of Medicine that could be repeatedly used for several learners. The supplies for this model and the setup are shown in Appendix A. Alternatively, the standard papaya model could also be used.[12] The equipment needed and the setup for the simulation session are shown in Appendix B.

Implementation

This educational session was split into two parts: a large-group session and hands-on practice. On the day of the simulation, all participants gathered in a single classroom for the introductory, 30-minute, educational lecture and video demonstration. The lecture summarized the indications, contraindications, and steps for performing MUA, as well as possible complications of the procedure (Appendix C). A video contained within this slide deck demonstrated the steps of MUA in the correct order as performed on the simulation model (Appendix D).

Following the lecture and video demonstration, the larger group was divided into four smaller groups of seven to eight learners each. Participant subgroups then took turns rotating through the hands-on learning portion in the simulation center while the remaining subgroups participated in other planned educational activities as part of the overall EM simulation day.

Copyright © 2024 Wegman et al. This is an open-access publication distributed under the terms of the Creative Commons Attribution license.

The hands-on learning portion of the instruction contained three stations (Figure 1). Stations 1 and 2 consisted of the critical steps for performing MUA that were considered novel for the EM learner and were allotted 10 minutes for each learner. Station 1 was paracervical and cervical anesthesia, and station 2 was cervical dilation. For each of these stations, there were two models. Stations 1 and 2 could be completed in either order, but both needed to be completed to move to station 3. Station 3 provided the opportunity to practice all steps for MUA in order. This was to allow for deliberate practice of the first two steps and then situate the whole procedure within mastery learning with the stepwise progression of difficulty. For this complete MUA station, the uterine model was situated inside a hemipelvic model to simulate the psychomotor skills needed for the procedure and a focus on sterile technique. Station 3 consisted of four hemipelvic models. We allotted 15 minutes per learner for this station. To account for transition time between stations and groups, we allotted 45 minutes total to each subgroup's hands-on portion of the session.

We recruited five board-certified gynecology faculty facilitators for the planned instructional session. Two to three gynecology facilitators started at either station 1 or 2, guiding three participants at a time through the initial steps of the MUA procedure. Each participant completed stations 1 and 2 on their own individual model. As participants finished stations 1 and 2, facilitators pivoted to a pelvic model at station 3 and supervised one learner at a time as they completed the entire MUA procedure from start to finish.

Facilitators were given facilitator guides (Appendix E) highlighting critical steps to be completed at each station. Using these guides, they provided verbal cues and feedback to learners as they completed each station. Learners were kept at a station until they were able to articulate key components of that step to the facilitator and successfully complete the procedures as indicated in the facilitator guide. At station 3, facilitators used a learner checklist (Appendix F) to ensure that all key steps of the entire MUA procedure had been performed in order by each learner on the pelvic model and that the learner was able to articulate indications for the procedure and explain when the procedure would be higher risk. The facilitator guide and checklist were created with our gynecology faculty based on their expertise and experience.

Evaluation

Anonymous course evaluations collected quantitative and qualitative data. A single retrospective pre-post self-assessment,[13] administered at the completion of the education sessions, assessed learners' self-reported confidence and comfort regarding aspects of MUA prior to and after the session on 5-point Likert scales (Appendix G). We also queried learners'

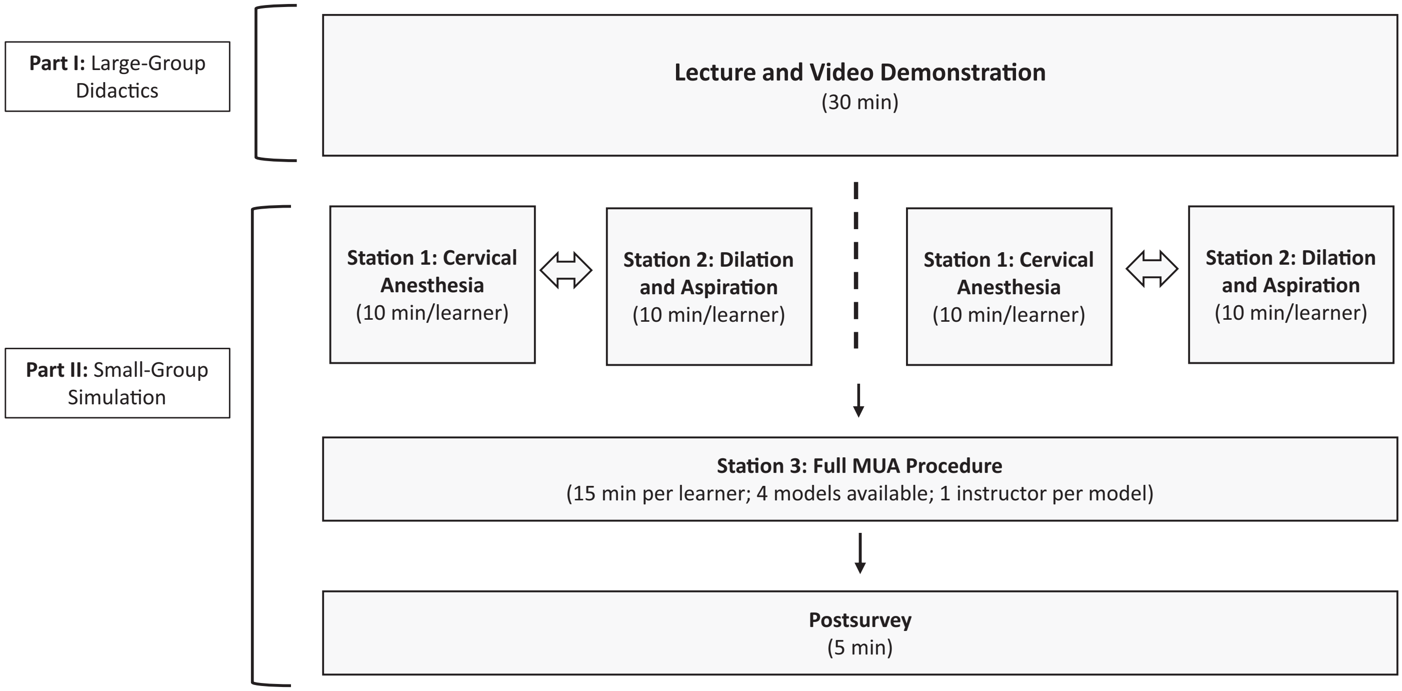

Figure 1. Structure of educational session. Abbreviation: MUA, manual uterine aspiration.

Copyright © 2024 Wegman et al. This is an open-access publication distributed under the terms of the Creative Commons Attribution license.

self-reported likelihood of considering MUA in the ED and their interest in further education. The data from all learners were pooled based on the assumption that this was a novel procedure for all. Finally, to evaluate the curriculum, participants were asked about the perceived effectiveness of the content and given space for open-ended feedback. We also sought feedback from the faculty facilitators (Appendix H).

Results

Thirty EM learners participated in the MUA simulation curriculum, of whom 23 were EM residents (seven PGY 1s, six PGY 2s, four PGY 3s, and six PGY 4s), two pediatric EM fellows, and five fourth-year medical students on their EM clinical rotation. We assessed both self-reported confidence in learners' ability to identify indications and high-risk scenarios for MUA and comfort in ability to perform various steps of the procedure using 5-point Likert scales (1 = *not at all confident/very uncomfortable,* 5 = *extremely confident/very comfortable*). We had a 100% response rate from the workshop participants.

Participant responses regarding comfort with identifying indications for MUA as well as high-risk scenarios for the procedure are displayed in Figure 2. All participants reported increased comfort for both items. Prior to the session, no participant reported that they were quite or extremely confident in their ability to identify indications for MUA compared to 80% (24 of 30 participants) posttraining. Similarly, no participant felt quite or extremely confident in their ability to identify cases that would be deemed higher risk for MUA in the ED prior to the session compared to 73% (22 of 30 participants) after the session.

Additionally, all participants had increased comfort in performing the individual steps as well as the complete ED MUA procedure after completion of the simulation training (Figure 3). All respondents but one were very uncomfortable in performing MUA prior to this session, while 70% (21 of 30 participants) felt comfortable or very comfortable with performing the procedure after the session. The majority of participants (23 of 30) reported that they were likely or very likely to consider MUA in the appropriate clinical circumstance; the same number also indicated that they were interested or very interested in further education on MUA and its potential application within the EM physician's scope of practice.

Utilizing the procedural checklist (Appendix F), the gynecology faculty facilitators actively observed all learners (30 of 30) complete a simulated MUA from start to finish. Prior to their completing the simulated MUA, each participant was asked to describe the indications for MUA and potential higher-risk scenarios to the facilitator and successfully did so. They were then able to demonstrate their ability to successfully perform all the integral steps of MUA, including a paracervical block, cervical dilatation, and finally the full MUA procedure, to complete the training.

Written comments from learners emphasized the effectiveness of the stepwise progression model of teaching. They valued the opportunity to learn the individual steps of the MUA procedure

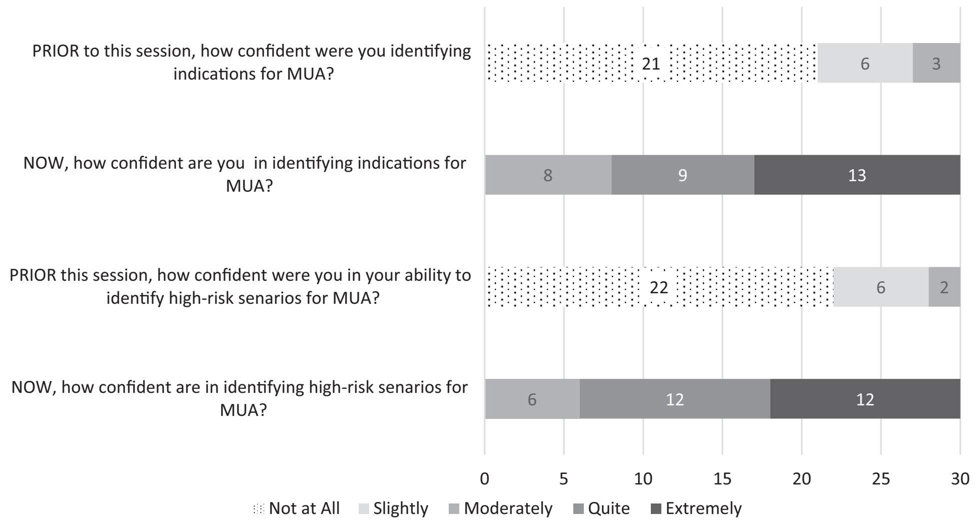

Figure 2. Participants' survey responses regarding confidence identifying indications for MUA and potential high-risk scenarios ($N = 30$). Abbreviation: MUA, manual uterine aspiration.

Copyright © 2024 Wegman et al. This is an open-access publication distributed under the terms of the Creative Commons Attribution license.

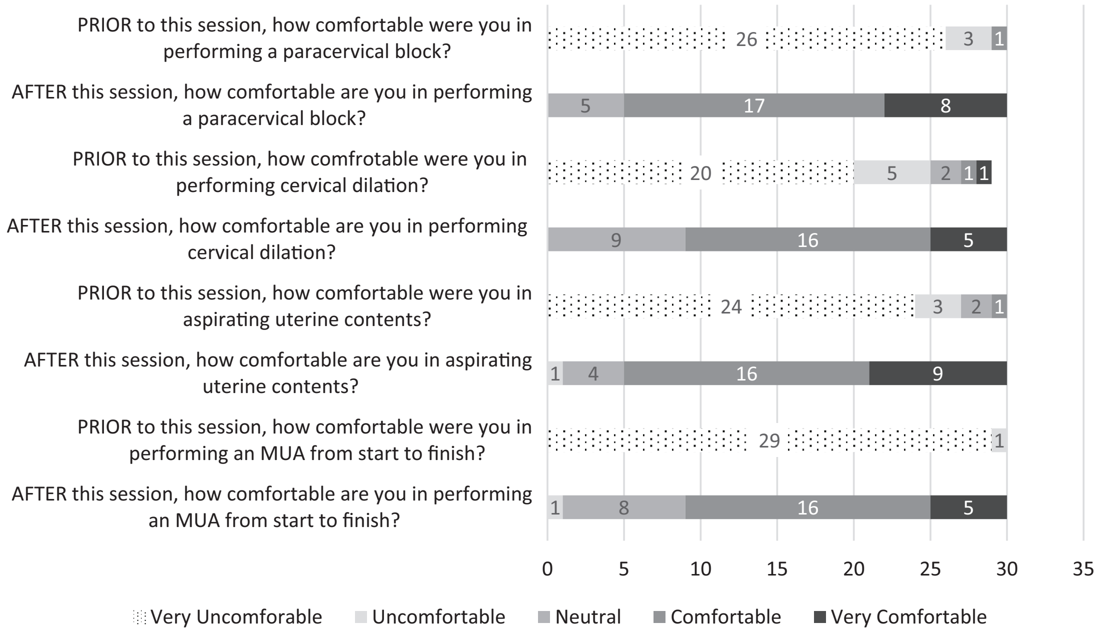

Figure 3. Participants' survey responses regarding comfort performing the steps of MUA ($N = 30$). Abbreviation: MUA, manual uterine aspiration.

before putting them all together for the complete procedure. Participants also commented on the value of having gynecology faculty facilitators available to teach them and respond to their nuanced questions during the simulation session. Multiple participants expressed that they were surprised they had never heard of the procedure before and were not familiar with its use in the ED. All five facilitators felt that overall, the curriculum and the simulation model were extremely effective, and all 30 learners rated the model as effective or highly effective. Comments from faculty highlighted the quality and effectiveness of the model as well as its ability to better recreate the tactile feel of this procedure, particularly during cervical dilation and aspiration. They also noted that some learners struggled with the uterine aspirator but felt that the stations were well timed and allowed learners to adequately progress through the individual skills stations before performing the MUA procedure in totality.

Discussion

We created a unique curriculum to train EM learners to perform MUA in the ED. EM skills focus on care and stabilization of the acutely ill or injured patient. While MUA is the definitive management of hemorrhagic complications of EPL, it is not included in most EM training programs. As a result, while hemorrhagic complications of EPL are rare, EM physicians are limited in their ability to definitively manage and obtain source control. This curriculum was well received by both learners and facilitators. In particular, learners expressed increased confidence in identifying indications and high-risk scenarios for the procedure. This is of potential benefit for those who practice in communities where access to specialty obstetrics and gynecologic care may not be readily available, including austere or global health settings or within the increasing number of maternal care deserts in the United States.[14]

We believe the curriculum utilized an effective MUA model to prepare the EM provider for this rare but potentially lifesaving procedure when compared to the typical papaya model of learning MUA. While a papaya fruit has previously been used as an introduction to MUA for learners and offers an easily implementable and low-cost model,[12] the model used in this curriculum confers several advantages. The pool noodle model can be refilled with simulated uterine contents multiple times and used repeatedly for multiple learners over a longer time period. As highlighted by our gynecologic facilitators' responses, this model also offers higher fidelity, with the elastic cervix providing a more realistic sense of the tactile resistance met during dilation and aspiration, and the Velcro interior of the model results in a gritty texture similar to what would be felt near the end of the procedure by a provider performing MUA.

Despite testing this session with novice learners before its full implementation, we were surprised to learn that the use of the manual uterine aspirator (the Ipas model) required more time and specific instruction than anticipated. Engaging the vacuum was not intuitive and required frequent assistance from the facilitators.

Copyright © 2024 Wegman et al. This is an open-access publication distributed under the terms of the Creative Commons Attribution license.

In future sessions, we would add more explicit instructions on the uterine aspirator to station 2 and slightly more time at this station.

Limitations of the curriculum include evaluation data that focused primarily on self-reported comfort and confidence and did not necessarily translate to ability to perform the procedure on a patient. As this is a novel procedure for most EM learners, our initial goals centered on the feasibility of such a curriculum, the introduction of this new procedure to novice learners, and increased learner self-efficacy. We do not have data on skills retention after this training. As with other high-acuity, low-frequency procedures taught in EM, this would likely require more frequent simulation practice.[15] We would anticipate the need for additional training to assess both knowledge and skill retention and to continue skill development and refinement within the framework of deliberate practice.[9] Overall, this session was well received by learners and is an opportunity to expand EM education and knowledge about the application of MUA in the ED to benefit patients, communities, and the health care system.

Appendices

A. MUA Model Preparation.docx

B. Station Setup and Supplies.docx

C. MUA Lecture.pptx

D. MUA Video Demonstration.m4v

E. Facilitator Guides.docx

F. Procedure Checklist.docx

G. Learner Survey.docx

H. Facilitator Survey.docx

All appendices are peer reviewed as integral parts of the Original Publication.

Katherine Wegman, MD: Fourth-Year Resident, Department of Emergency Medicine, Boston Medical Center

Caroline Gorka, MD: Fourth-Year Resident, Department of Emergency Medicine, Boston Medical Center

Judith Linden, MD: Professor, Department of Emergency Medicine, Boston University Chobanian & Avedisian School of Medicine; ORCID: https://orcid.org/0000-0002-2677-3410

Shannon Bell, MD: Assistant Professor, Department of Obstetrics and Gynecology, Boston University Chobanian & Avedisian School of Medicine; ORCID: https://orcid.org/0000-0002-8141-5122

Stephanie N. Stapleton, MD: Assistant Professor, Department of Emergency Medicine, Boston University Chobanian & Avedisian School of Medicine; ORCID: https://orcid.org/0000-0003-1593-1943

Virginia Tancioco, MD: Attending Physician, Department of Obstetrics and Gynecology, Roseville Medical Group; ORCID: https://orcid.org/0000-0003-4300-6253

Laura Welsh, MD: Assistant Professor, Department of Emergency Medicine, Boston University Chobanian & Avedisian School of Medicine; ORCID: https://orcid.org/0000-0002-3987-2186

Disclosures
None to report.

Funding/Support
None to report.

Ethical Approval
Reported as not applicable.

References

1. ACOG Practice Bulletin no. 200: early pregnancy loss. *Obstet Gynecol*. 2018;132(5):e197-e207. https://doi.org/10.1097/AOG.0000000000002899
2. Wittels KA, Pelletier AJ, Brown DFM, Camargo CA Jr. United States emergency department visits for vaginal bleeding during early pregnancy, 1993-2003. *Am J Obstet Gynecol*. 2008;198(5): 523.e1-523.e6. https://doi.org/10.1016/j.ajog.2007.11.011
3. Benson LS, Magnusson SL, Gray KE, Quinley K, Kessler L, Callegari LS. Early pregnancy loss in the emergency department, 2006–2016. *J Am Coll Emerg Physicians Open*. 2021;2(6): e12549. https://doi.org/10.1002/emp2.12549
4. Whittaker L, Pymar H, Liu XQ. Manual uterine aspiration in the emergency department as a first-line therapy for early pregnancy loss: a single-centre retrospective study. *J Obstet Gynaecol Can*. 2022;44(6):644-649. https://doi.org/10.1016/j.jogc.2022.02.009
5. McCoy EE, Weidner AKH, Benson LS, Darney BG, Prager SW. Practice change following an interdepartmental training on early pregnancy loss management in the emergency department. *Cogent Public Health*. 2022;9(1):2052402. https://doi.org/10.1080/27707571.2022.2052402
6. Quinley KE, Chong D, Prager S, Wills CP, Nagdev A, Kennedy S. Manual uterine aspiration: adding to the emergency physician stabilization toolkit. *Ann Emerg Med*. 2018;72(1):86-92. https://doi.org/10.1016/j.annemergmed.2017.10.019
7. Kinariwala M, Quinley KE, Datner EM, Schreiber CA. Manual vacuum aspiration in the emergency department for management of early pregnancy failure. *Am J Emerg Med*. 2013;31(1): 244-247. https://doi.org/10.1016/j.ajem.2012.07.014
8. Farrell RG, Stonington DT, Ridgeway RA. Incomplete and inevitable abortion: treatment by suction curettage in the emergency department. *Ann Emerg Med*. 1982;11(12):652-658. https://doi.org/10.1016/S0196-0644(82)80256-4

Copyright © 2024 Wegman et al. This is an open-access publication distributed under the terms of the Creative Commons Attribution license.

9. Ericsson KA. Deliberate practice and acquisition of expert performance: a general overview. *Acad Emerg Med*. 2008; 15(11):988-994. https://doi.org/10.1111/j.1553-2712.2008.00227.x

10. McGaghie WC, Issenberg SB, Cohen ER, Barsuk JH, Wayne DB. Does simulation-based medical education with deliberate practice yield better results than traditional clinical education? A meta-analytic comparative review of the evidence. *Acad Med*. 2011;86(6):706-711. https://doi.org/10.1097/ACM.0b013e318217e119

11. McGaghie WC, Issenberg SB, Barsuk JH, Wayne DB. A critical review of simulation-based mastery learning with translational outcomes. *Med Educ*. 2014;48(4):375-385. https://doi.org/10.1111/medu.12391

12. Steinauer J, Preskill F, Robertson P. Training medical students in intrauterine procedures using papayas. *Med Educ*. 2007;41(11): 1099-1100. https://doi.org/10.1111/j.1365-2923.2007.02878.x

13. Bhanji F, Gottesman R, de Grave W, Steinert Y, Winer LR. The retrospective pre–post: a practical method to evaluate learning from an educational program. *Acad Emerg Med*. 2012;19(2): 189-194. https://doi.org/10.1111/j.1553-2712.2011.01270.x

14. Wallace M, Dyer L, Felker-Kantor E, et al. Maternity care deserts and pregnancy-associated mortality in Louisiana. *Womens Health Issues*. 2021;31(2):122-129. https://doi.org/10.1016/j.whi.2020.09.004

15. Wang EE, Quinones J, Fitch MT, et al. Developing technical expertise in emergency medicine—the role of simulation in procedural skill acquisition. *Acad Emerg Med*. 2008;15(11): 1046-1057. https://doi.org/10.1111/j.1553-2712.2008.00218.x

Received: November 7, 2023
Accepted: August 16, 2024
Published: November 11, 2024

Copyright © 2024 Wegman et al. This is an open-access publication distributed under the terms of the Creative Commons Attribution license.
